# Nanoemulsion-loaded hydrogel coatings for inhibition of bacterial virulence and biofilm formation on solid surfaces

**DOI:** 10.1038/s41598-019-43016-w

**Published:** 2019-04-24

**Authors:** Prateeksha, Saroj Kanta Barik, Brahma Nand Singh

**Affiliations:** Herbal Nanobiotechnology Lab, Pharmacology Division, CSIR-National Botanical Research Institute, Lucknow, 226001 India

**Keywords:** Antimicrobial resistance, Drug development

## Abstract

The indiscriminate use of antibiotics has led to the emergence of drug-resistant bacteria which has become one of the biggest challenges of the twenty-first century for the researchers to combat and in turn search for novel targets which could lead to the development of effective and sustainable therapies. Inhibition of biofilm formation and virulence of bacterial pathogens is an emerging approach to address the challenges related to bacterial infections. To suppress the virulence and biofilm formation by *Escherichia coli* O157:H7 (ECOH), we developed stable nanoemulsion (NE) of *Gaultheria fragrantissima* Wall. essential oil’s (EO) bioactive compounds, viz., eugenol (E-NE) and methyl salicylate (MS-NE) that showed significantly higher anti-biofilm and anti-virulence activities as compared to eugenol and methyl salicylate without affecting ECOH planktonic cell growth. Transcriptional analysis showed that E-NE and MS-NE reduced the expression of genes, including curli, type I fimbriae, Shiga-like toxins, quorum sensing, and *ler*-controlled toxins, which are needed for biofilm formation, pathogenicity, and attachment. E-NE and MS-NE loaded hydrogel coatings showed superior anti-biofilm activity against ECOH on glass, plastic and meat surfaces as compared to eugenol and methyl salicylate loaded coatings. Conclusively, NE-loaded hydrogel coatings could be used in combating ECOH infection on solid surfaces through anti-biofilm and anti-virulence strategies.

## Introduction

*Escherichia coli* O157:H7 (ECOH) is one of the Shiga toxin–producing serotype that causes hemorrhagic colitis, bloody diarrhea, kidney failure, and abdominal cramps^1^. ECOH is commonly found in the large intestine of mammals, and could be ingested through undercooked meat, colonized surfaces, and contaminated water^1,2^. More specifically, Shiga-like toxin is associated with hemolytic-uremic syndrome^3^. ECOH can survive in adverse conditions including low-moisture environments and also has the capability to develop resistance to antibiotic^4^. There is no effective therapy available due to the failure of existing antimicrobial agents and the emergence of drug-resistant strains. Also increased dosage causes the hemolytic-uremic syndrome, a common factor of acute kidney injury in children^3^.

Bacterial biofilms are amorphous and dynamic mono- or poly-microbial structures adhering to living or non-living surfaces^5^. This adaptation is a common survival strategy used by bacteria to shield from antimicrobial agents and also provides resistance from host immune clearance. To develop biofilm, bacterial pathogens produce an extracellular polymeric substance composed of proteins, nucleic acids, polysaccharides, and water as the main components^5,6^. The biofilm formation enhances the pathogenicity of bacteria and plays a key role in causing nosocomial infections^5^.

The ability of ECOH to develop biofilm is the most important virulence characteristic that increases its survival in harsh conditions. Biofilms are multiple component structures of bacteria and some fungi, particularly *Candida albicans* in which cells stick to each other on living or non-living surfaces^7–12^. Biofilms protect bacteria from the treatment of antibiotics and external aggregation such as predator attacks. ECOH has also the ability to form antibiotic-resistant biofilms on solid surfaces, including, plastic, glass, and meat^13^. Biofilm formation in ECOH has implications in mortality, lipopolysaccharide (LPS) secretion, and fimbriae production^14,15^. Because ECOH is a clinically important pathogen, search for potent biofilm inhibitors from phytosources without affecting the bacterial cell viability is urgently required which can also minimize the risk of drug resistance.

Essential oils (EOs) are plant-derived volatile secondary metabolites. Due to its strong antiseptic potential, EOs have been used since ancient times for treating microbial infections and other illnesses^13,16^. *Gaultheria fragrantissima* Wall. (Ericaceae) is an evergreen perennial shrub widely distributed in the Himalayas and the northeastern region of India^17^. The EO of leaves contains volatile organic ester, methyl salicylate that can serve as an effective natural source of the commonly used pain reliever, aspirin^18^. However, due to its volatile nature, low bioavailability and high degradation rate, the real application of *G*. *fragrantissima* EOs is limited.

In recent years, nanomaterials are emerging as efficient tools to deliver lipophilic drugs and molecules^19–24^. Among them, nanoemulsion (NE) of EOs with suitable delivery systems is an appropriate strategy to overcome these obstacles. NEs are a mixture of oil and water phases, stabilized with surfactants and has a particle size of less than 200 nm^25^. Oil in water (O/W) type NEs are excellent carriers for delivery of EOs because of their potential to solubilize large quantity of EOs and to protect them from evaporation, hydrolysis, and degradation^26,27^. Moreover, the fabrication of antimicrobial hydrogels using NEs becomes an emerging technology in recent years. Hydrogels are a promising class of materials fabricated from natural or synthetic polymers that display 3D network structures with high to ultra-high degree of water content^28–31^. Due to their high hydrophilicity, complex three-dimensional network, unique biocompatibility, and cell adhesion, hydrogels are one of the suitable biomaterials for the development of surface coatings in preventing and treating multidrug-resistant infections^19^. Currently, there is a great deal of research going on in the area of developing antimicrobial hydrogel-based coatings of plant-derived phytochemicals and EOs. The EO of *G*. *fragrantissima* has been reported to be having insecticidal, nematicidal, antioxidant and antibacterial activities^32^. However, anti-biofilm and anti-virulence properties of its EO and bioactive compounds have not been reported so far. Therefore, there was a specific interest in developing NE-loaded hydrogel coatings of bioactive compounds of *G*. *fragrantissima* EO for antimicrobial application.

## Results

### Characterization of NEs

**Figure 1:**
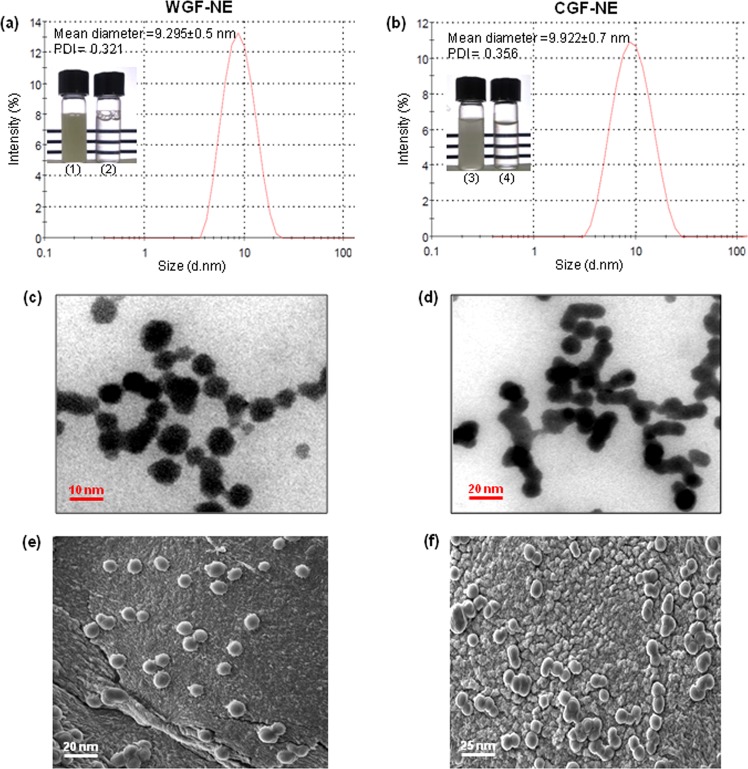
Characterization of NEs. DLS particle size distribution of (**a**) WGF-NE and (**d**) CGF-NE. The insets depict the emulsion of WGF-EO (1), WGF-NE (2), emulsion of CGF-EO (3) and CGF-NE (4). SEM images of (**c**) WGF-NE and (**e**) CGF-NE. TEM images of (**c**) WGF-NE and (**f**) CGF-NE.

O/W type NEs were prepared from EO of wild *G*. *fragrantissima* (WGF) leaves and commercial grade EO of *G*. *fragrantissima* (CGF) using Tween-80 (surfactant), propylene glycol (co-surfactant), and distilled water (DW). The volumes of EO (5%), the non-ionic surfactant and emulsifier (S) (Tween 80; 0.2%), co-surfactant (CS) (propylene glycol; 0.1%) and DW in the ratio of 5(EO):0.2(S):0.1(CS):94.7(DW) were used to prepare the NE. The mixture was sonicated using a 20 kHz sonicator with a maximum power output of 750 W for 20 min while maintaining the sample in an ice-bath. The mean droplet diameter of 9.295 ± 0.5 nm (Fig. 1a) and 9.922 ± 0.7 nm (Fig. 1b) with a polydispersity index (PDI) of 0.321 and 0.356 of WFG-NE and CGF-NE, respectively was measured by Litesizer-500 particle size analyzer (Anton Paar, Australia). The PDI less than 0.4 indicated that the NE droplets were mono-dispersed. To determine the shape and morphology of the prepared NEs, transmission electron microscopy (TEM) was performed. The obtained NEs were spherical in shape with a particle size ranging from 5–15 nm in diameter for WGF-NE (Fig. 1c) and 6–16 nm for CGF-NE (Fig. 1d). The average particle size was also determined by scanning electron microscopy (SEM) and it was found to be 8.3 ± 1.2 nm for WGF-NE (Fig. 1e) and 19.1 ± 2.82 for CGF-NE (Fig. 1f). The prepared WGF-NE was stable even after being subjected to centrifugation for 20 min at 10,000 rpm at room temperature. WGF-NE was found to be more stable when stored at 4 °C, −20 °C and 45 °C as compared to CGF-NE. Both phase separation and creaming were not detected when stored at room temperature for 90 days (Table S1).

### Minimum inhibitory concentration (MIC) and sub-MIC

**Figure 2:**
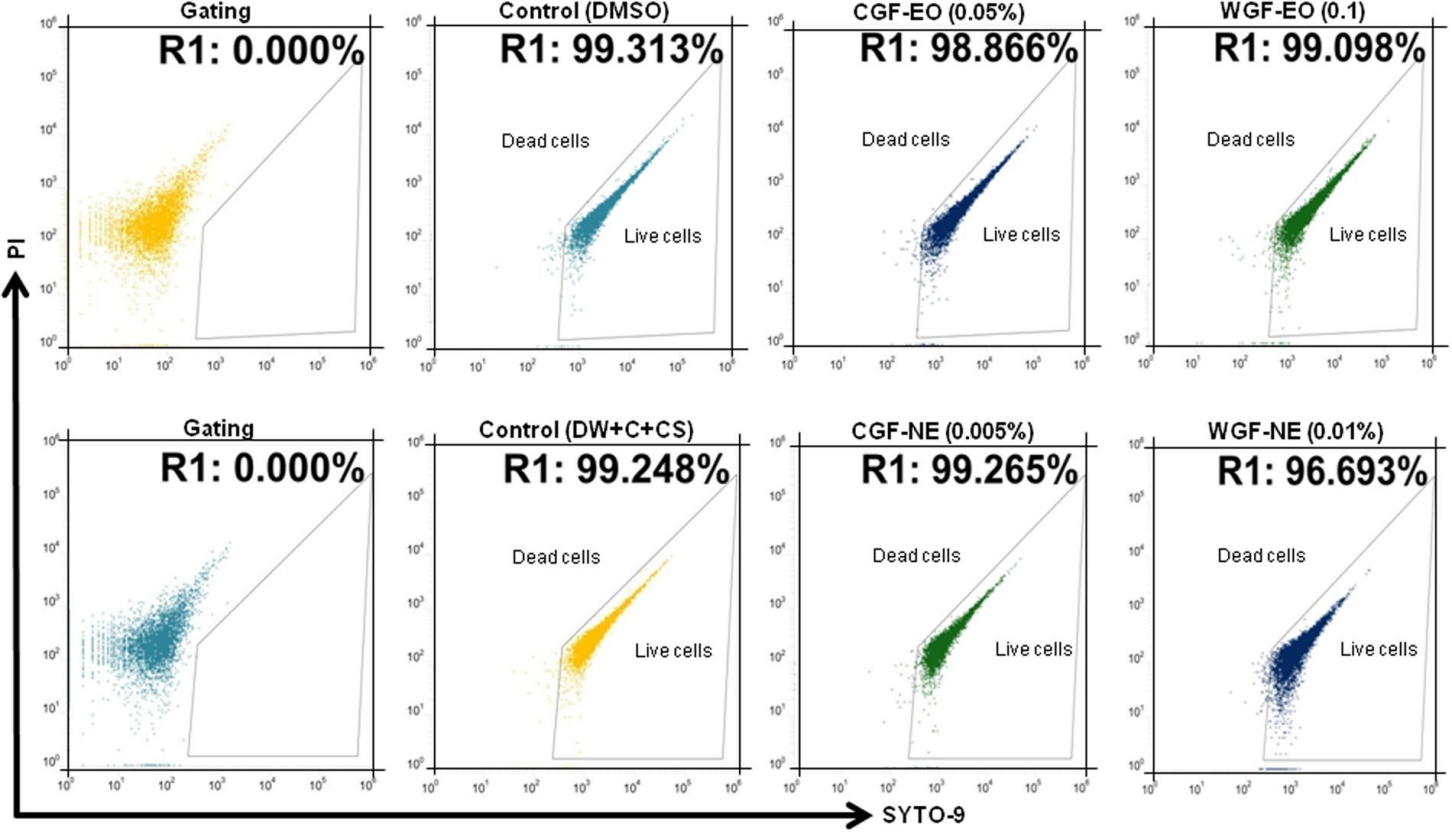
Sub-MIC and MIC determination against ECOH using fluorescence microscopy. Cells were exposed to the indicated samples with indicated concentrations for 24 h and analysed by flow cytometry using live/dead bacterial cell viability kit. DMSO was used as a solvent control for CGF-EO and WGF-EO, whereas the mixture of DW, surfactant (S; Tween 80) and co-surfactant (CS; propylene glycol) in the ration of 94.7:0.2:0.1 was used as a solvent control for CGF-NE and WGF-NE.

MICs of EOs and NEs were determined against ECOH using growth kinetics assay. MIC was evaluated as the lowest concentration that exhibited entire suppression of the visible growth. The MIC values of CGF-EO and CGF-NE were 0.5% and 0.1%, respectively. While, WGF-EO and WGF-NE exhibited MIC at the concentration of 0.3% and 0.025%, respectively (Table S2). Next, sub-MIC levels of the test samples were determined by flow cytometry and bacterial growth curve analyses. When the cells were exposed to 0.1% of CGF-EO and 0.01% of CGF-NE, no significant reduction of ECOH cell viability was determined (Fig. 2). Moreover, WGF-EO at 0.05% and WGF-NE at 0.005% did not show any cidal effects (Fig. 2). Similar results were also recorded in the growth curve analysis (Fig. S1a–d). Cell viability analysis was performed using a fluorescence microscope to further confirm the MIC and sub-MIC values of WGF-EO and WGF-NE. Cell death was not observed when ECOH treated with sub-MIC levels (Fig. S2). Moreover, sub-MIC values of CGF-EO and CGF-NE were also not found to induce cell death (data not shown). The sub-lethal concentrations would not possibly enforce a discriminating pressure to elevate drug resistance in ECOH. Hence, sub-lethal concentrations of CGF-EO, CGF-NE, WGF-EO, and WGF-NE were selected for anti-biofilm and anti-virulence experiments.

### Anti-biofilm Potential of EOs and NEs

**Figure 3:**
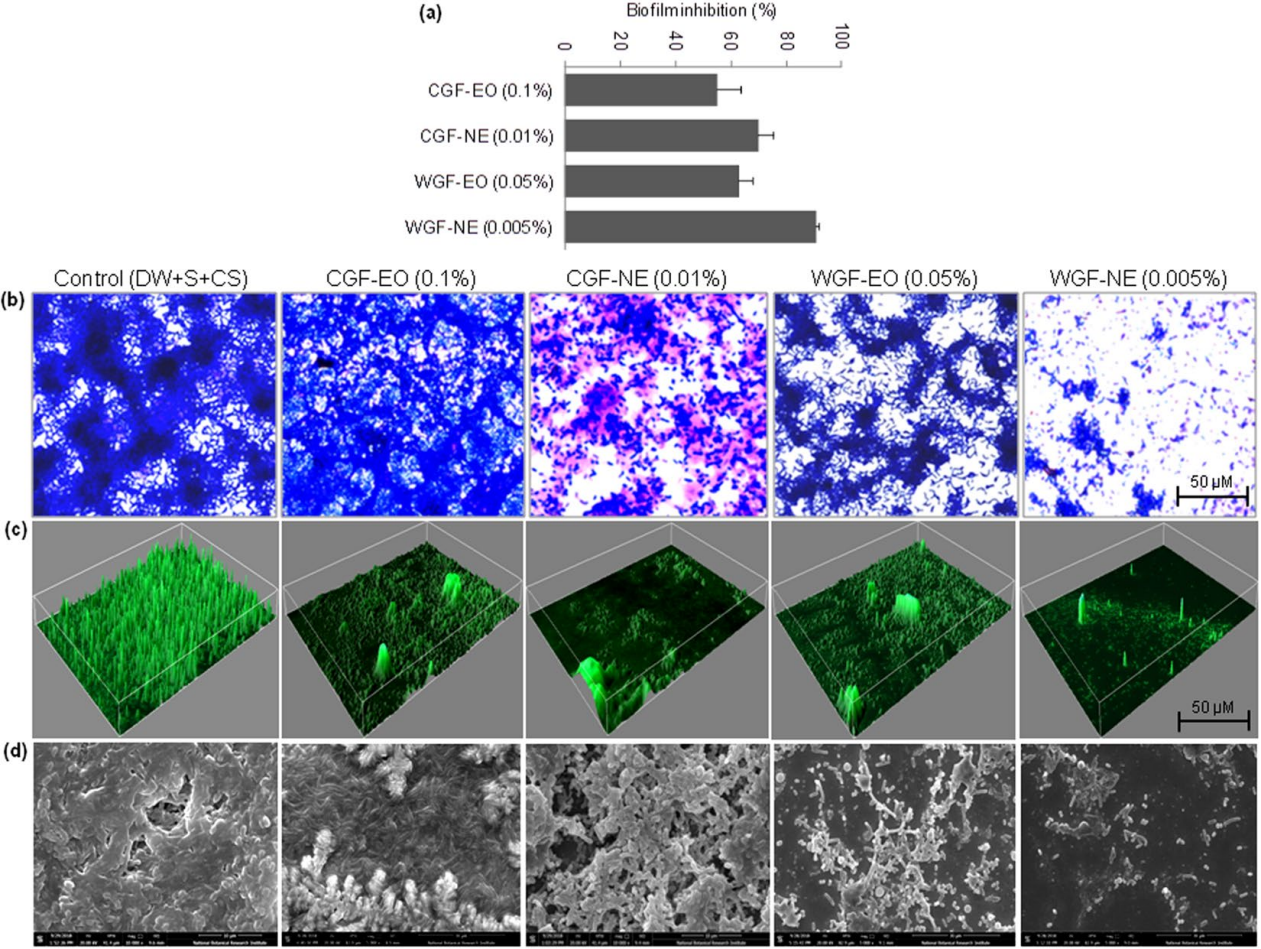
Anti-biofilm potential of EOs of *G*. *fragrantissima* and their NEs against ECOH. Inhibition of biofilm in the presence of EOs and NEs was measured after 24 h treatment. The results were expressed as arithmetic mean ± SE. The experiments were carried out at least thrice in independent experiments having three replicates. (**a**) ELISA assay in 96-well plates, (**b**) CV-stained cell imaging by phase contrast microscope, (**c**) SYTO/PI stained cell imaging by confocal laser microscopy and (**d**) SEM imaging. The mixture of DW, surfactant (S; Tween 80) and co-surfactant (CS; propylene glycol) in the ration of 94.7:0.2:0.1 was used as a solvent control.

To examine anti-biofilm potential of CGF-EO, CGF-NE, WGF-EO, and WGF-NE at sub-lethal concentrations were initially quantified in 96-well polystyrene plates using crystal violate (CV) staining ELISA assay (Fig. 3a). All the samples showed an inhibitory effect on biofilm formation against ECOH. However, the highest potential was exhibited by WGF-NE (91%), followed by CGF-NE (70%), WGF-EO (63%) and CGF-EO (55%). Similarly, the anti-biofilm activity of test samples was also observed using CV-stained biofilms under a phase contrast microscope (Fig. 3b).

In the current investigation, it was also observed that the CGF-EO, CGF-NE, WGF-EO, and WGF-NE inhibited ECOH biofilm formation on glass coverslips which was analyzed by fluorescence microscope. ECOH forms biofilms at the bottom and on the sides of the wells. ECOH stained with green fluorescent dye SYTO-9 was used to analyze biofilm formation. The microscopic observations established that WGF-NE had the highest inhibitory effect on the biofilm formation (Fig. 3c). The anti-biofilm activity of EOs and NEs was further confirmed by SEM (Fig. 3d). Anti-biofilm potential was also validated by COMSTAT analysis. WGF-NE and CGF-NE decreased biomass, mean thickness, and substratum coverage of ECOH. Biomass in terms of volume/area and mean thickness were decreased by 91% and 69% by WGF-0.005% NE and 0.01% CGF-NE, respectively. However, no anti-biofilm activity was observed, when ECOH cells treated with the same concentrations of WGF-EO and CGF-EO (Table 1).

**Table 1:** Anti-biofilm activity of EOs and NEs against ECOH using COMSTAT analysis.

Treatment	Volume/Area (μm^3^ μm^−2^)	Mean thickness (μm)	Substratum coverage (%)
Control	17.1 ± 2.7	15.6 ± 1.83	82 ± 3.74
CGF-EO (0.01%)	17.1 ± 2.5	15.4 ± 2.1	82 ± 5.71
CGF-NE (0.01%)	5.3 ± 0.27	3.7 ± 0.31	10 ± 1.16
WGF-EO (0.005%%)	16.7 ± 2.66	15.0 ± 2.14	80 ± 4.62
WGF-NE (0.005%)	1.4 ± 0.25	1.2 ± 0.16	4 ± 0.31

Data represent mean ± SE of six different experiments.

### Anti-biofilm Bioactive Chemicals of Eos

To identify the bioactive chemical constituents of CGF-EO and WGF-EO, gas chromatography/mass spectroscopy (GC-MS) analysis was performed. EO from the leaves of wild *G*. *fragrantissima* was extracted using Clevenger apparatus and EO yield was 1.1%. GC–MS analysis of EOs showed the presence of 12 and 10 compounds in WGF-EO (Fig. S3a) and CGF-EO (Fig. S3b), respectively. The major chemical constituents of WGF-EO were methyl salicylate (98%), phenol 2-methoxy-3-2 propenyl (0.98%), and eugenol (0.6%). While methyl salicylate (>99%) and 2-hexenal (0.28%) were major compounds in CGF-EO (Table 2). The results are in accordance with that of previous studies, in which the EO of *G*. *fragrantissima* leaves has more than 93% methyl salicylate^33^. However, the current study reported for the first time having eugenol as a bioactive compound in WGF-EO. Recently, Kim and colleagues reported the presence of eugenol in bay, cinnamon bark, clove, and pimento berry. EOs that suppressed *E*. *coli* O157:H7 biofilm formation by >80% at the concentration of 0.005%^13^. As expected, eugenol was also found to inhibit 80% biofilm formation of ECOH, when cells treated with 0.005% concentration. However, methyl salicylate required 0.025% for the inhibition of 80% ECOH biofilm formation (Fig. S4a). In addition, the anti-biofilm activity of eugenol and methyl salicylate was further confirmed by CV staining assay and fluorescent microscopy. However, phenol 2-methoxy-3-2 propenyl and 2-hexanal did not exhibit anti-biofilm activity at the concentration of 0.05% (data are not shown). These results suggest that eugenol and methyl salicylate were mostly accountable for the anti-biofilm activity of *G*. *fragrantissima* EO.

**Table 2:** GC-MS analysis of WGF-EO and CGF-EO.

RT (min)	Compound name	Composition (%)^a^	
		WGF-EO	CGF-EO
8.16	Trans 3-hexen-1-ol	0.07	0.05
8.67	2-Hexenal	0.05	**0.28**
12.83	Beta-terpineol acetate	—	0.03
12.85	D-Limonene	—	0.03
13.89	Eucalyptol	0.05	—
16.76	Beta-linalool	0.02	0.01
17.33	2-Myristynonyl pantetheine	—	0.02
20.57	4-Terpineol	0.01	—
21.51	5,8,11-heptadecatrien-1-ol	0.02	0.01
23.59	Methyl salicylate	**97.85**	**99.38**
25.71	Ethyl salicylate	—	0.03
29.13	Phenol 2-methoxy-3-2 propenyl	**0.98**	—
29.40	Trans-m-propenyl guaicol	—	0.04
29.81	Beta-curcumene	0.08	—
30.84	7-Epi cis sesquisabinene	0.01	—
34.84	6-Epi shyobunol	0.01	—
35.31	Eugenol	**0.60**	—

RT, retention time (minutes).

Components present in EOs at greater than 0.2% are indicated by bold font.

^a^Percentages were calculated based on normalized FID peak areas.

The antimicrobial activity of eugenol and methyl salicylate was studied by measuring MIC and ECOH planktonic growth. The MICs of eugenol and methyl salicylate against ECOH were 0.05% and 0.25% (Table S3), which is in concordant with earlier published data^13^. Notably, MICs of eugenol and methyl salicylate were 10-times greater than the sub-MIC levels (0.005% and 0.025%) which are needed for anti-biofilm activity (Table S3). The results demonstrate decreased biofilm formation without affecting the cell viability and growth of ECOH by the EOs of *G*. *fragrantissima* and their bioactive compounds, eugenol, and methyl salicylate.

### Anti-biofilm Activity of Eugenol-NE and Methyl Salicylate-NE

**Figure 4:**
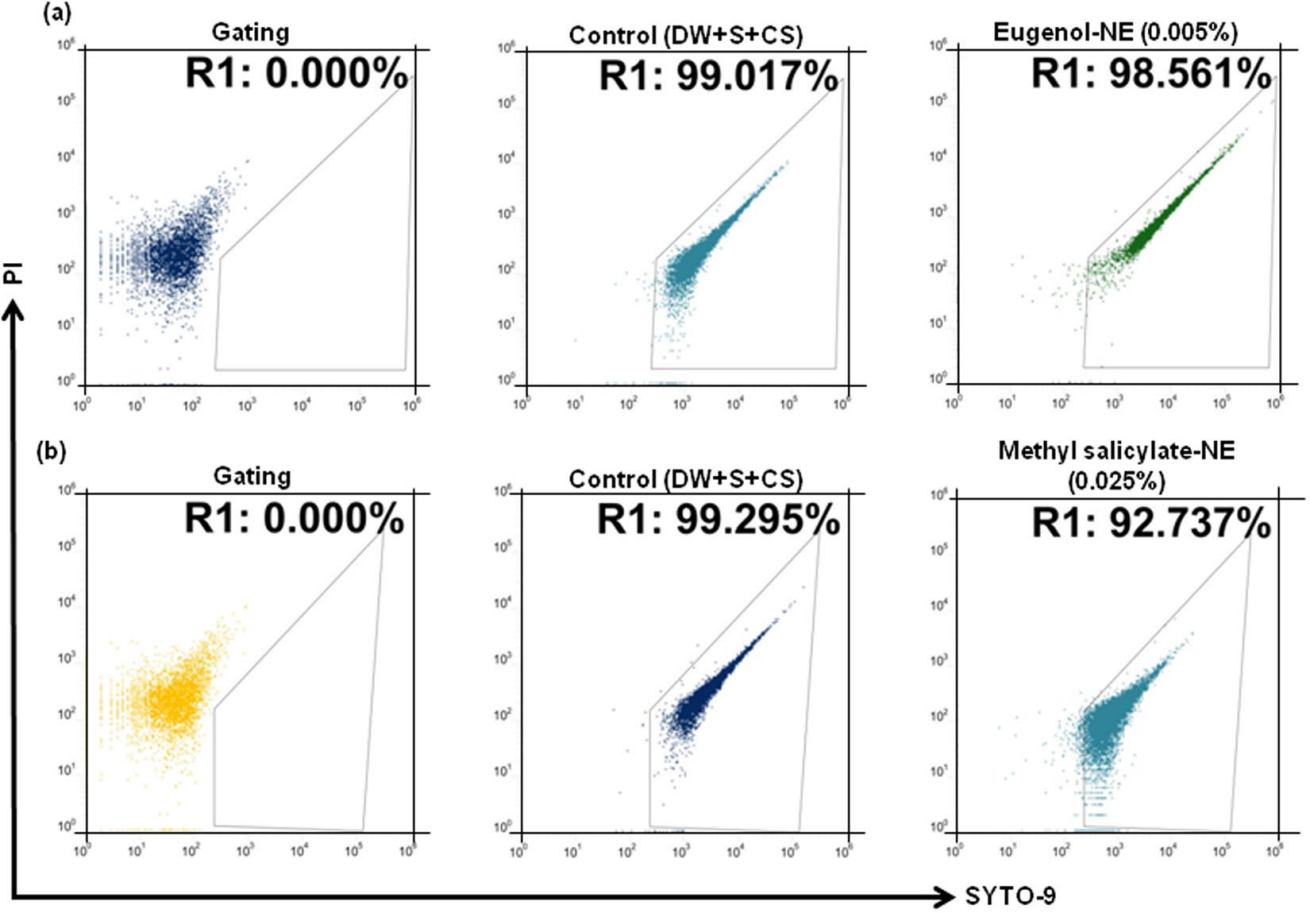
Determination of sub-MIC values of (**a**) eugenol-NE and (**b**) methyl salicylate-NE by flow cytometry using live/dead bacterial cell viability kit. Flow cytometry dot plots of ECOH cells treated with indicated samples for 24 h. The mixture of DW, surfactant (S; Tween 80) and co-surfactant (CS; propylene glycol) in the ration of 94.7:0.2:0.1 was used as a solvent control.

NEs of eugenol and methyl salicylate were prepared using an ultra-sonication method. The emulsion mixture contained water, compound, emulsifier polysorbate 80, and co-surfactant were mixed in the ratio of 94.7:5:0.2:0.1 (v/v). The mean droplet diameter of eugenol-NE and methyl salicylate-NE droplets were 9.389 ± 0.2 nm and 10.81 ± 0.4 nm and PDI 0.345 and 0.371, respectively (Fig. S5a,b). The droplet size measured using SEM was compared with the average droplet diameter measured by the particle size analyzer (Fig. S5c,d). Previous studies have found that NEs of various EOs have a spherical shape and 15–200 nm average droplet size^34^. Figure S5e,f showed brightfield microscopic images of NEs and these droplets were spherical in shape. The stability of prepared eugenol-NE and methyl salicylate-NE was also assessed. The prepared NEs were found to be highly stable when stored at 4 °C, −20 °C and 45 °C for a period of 90 days (data not shown). No alternations in droplet diameter were noticed. Moreover, neither phase separation nor creaming was recorded. MIC values of eugenol-NE and methyl salicylate-NE were 10-times lower than bulk eugenol and methyl salicylate (Table S4). Eugenol-NE and methyl-salicylate-NE exhibited sub-lethal values at the concentration of 0.0005% and 0.0025% respectively, measured by flow cytometry (Fig. 4a,b) and growth kinetics assay (Fig. S6a,b). Sub-lethal concentrations of eugenol-NE and methyl-salicylate-NE inhibited ECOH biofilm formation by 80% which was assessed by ELISA assay (Fig. S7a), fluorescence microscope (Fig. S7b), and phase contrast microscope (Fig. S7c). These sub-MIC levels of eugenol-NE and methyl salicylate were 10-times lower than their bulk forms. Interestingly, at the same concentration, eugenol and methyl salicylate did not inhibit biofilm formation of ECOH (Fig. S8a,b). These results confirmed that the process of nanotization enhances the anti-biofim activity of bioactive compounds of *G*. *fragrantissima* EO.

### Anti-virulence Activity of Eugenol-NE and Methyl Salicylate-NE

The production of LPS is a key phenotypic virulence factor of ECOH that play a key role in biofilm formation, architecture and maturation^35^. In the present study, LPS was fractionated from treated or untreated ECOH cultures and measured spectrophotometrically. The LPS production was reduced, when treated 0.0005% eugenol-NE and 0.0025% methyl salicylate-NE (Fig. S9a). Because motility plays a key role in *E*. *coli* biofilm formation, we also examined the impact of NEs on flagellar function through measurement of swimming and swarming motilities. Eugenol-NE (0.0005%) and methyl salicylate-NE (0.0025%) decreased swimming and swarming motilities of ECOH (Fig. S9b). The results were expressed as the diameter of the zone of inhibition. The data suggested that the anti-biofilm effect of eugenol-NE and methyl salicylate-NE on ECOH is related to motility suppression.

Fimbriae, a threadlike protein polymers are formed on the surface of ECOH that play an important role in biofilm formation and colonization of various specific host epithelia. Therefore, we assessed the effect of NEs on curli fimbriae formation by SEM and Congo red staining assay. Treatment of eugenol-NE (0.0005%) and methyl salicylate-NE (0.0025%) decreased fimbriae formation and specifically curli production, as evident from less accumulation of Congo red dye in NEs-treated cultures of ECOH as compared to untreated controls (Fig. S9c). However, at the same concentrations, bulk eugenol and methyl salicylate did not reduce the production of LPS, curli fimbriae formation, and motility in ECOH. The superior inhibitory effect of NEs over bulk eugenol and methyl salicylate might be due to their large surface area and small droplet size of NEs which permitted eugenol and methyl salicylate to interact more efficiently with fimbriae formation in ECOH cells.

### Effect of Eugenol-NE and Methyl Salicylate-NE on Virulence Genes Expression

**Figure 5:**
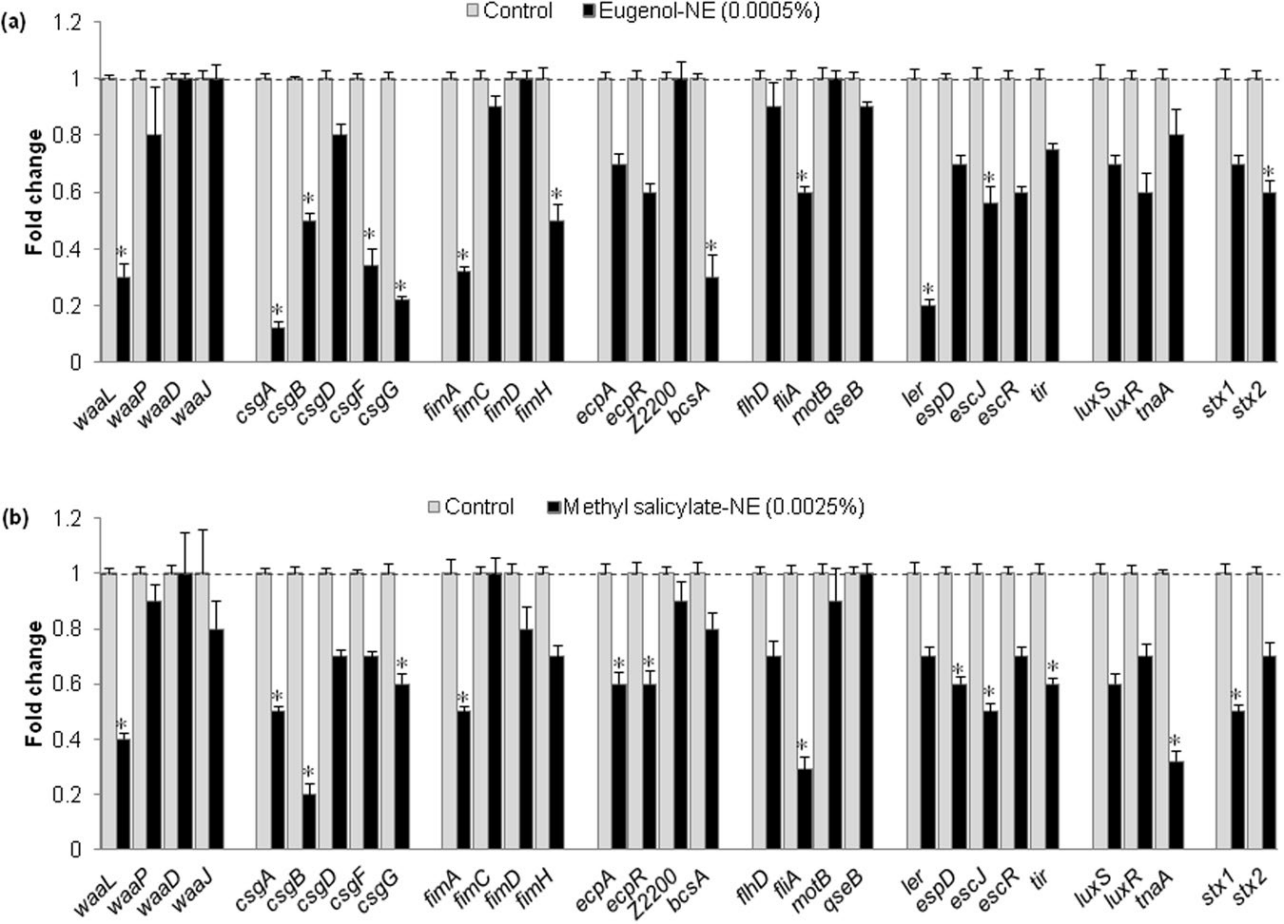
Transcriptional profiles of ECOH cells treated with or without (**a**) eugenol-NE and (**b**) methyl salicylate-NE for 24 h and expression of targeted genes was quantified by qRT-PCR. Relative gene expressions represent transcriptional levels after exposure to eugenol-NE and methyl salicylate-NE versus untreated controls (value 1.0). The mixture of DW, surfactant (S; Tween 80) and co-surfactant (CS; propylene glycol) in the ration of 94.7:0.2:0.1 was used as a solvent control. The data were expressed as arithmetic mean ± SE. The experiments were carried out at least thrice in independent experiments having three replicates. *P < 0.05 versus non-treated controls.

To explore the molecular basis of anti-biofilm activity of eugenol-NE and methyl salicylate-NE differential genes expression related to biofilm and virulence of ECOH was assessed using qRT-PCR. As shown in Fig. 5a,b, the targeted genes of ECOH were downregulated when treated with eugenol-NE (0.0005%) and methyl salicylate-NE (0.0025%), but these genes were not altered by the treatment of bulk eugenol and methyl salicylate. The genes related to biofilm formation, motility, toxin production, and quorum sensing were highly downregulated. Both eugenol-NE and methyl salicylate-NE reduced the expression of LPS biosynthesis (*waaL*, *waaP*, *waaD* and *waaJ*), swarming (*fimA* and *fimH*), swimming (*flhD*, *fliA*, and *motB*), LEE transcriptional regulator (*ler*), *ler* and *ler*-regulated toxin (*espD*, *escJ*, *escR* and *tir*), quorum sensing (*luxS*, *luxR* and *tnaA*) and Shiga-like toxin (*stx1* and *stx2*) genes. Moreover, qRT-PCR results revealed that the NEs of eugenol and methyl salicylate strongly reduces the expression of virulence-related genes of ECOH. However, NEs did not alter the expression of a housekeeping gene, *rrsG*.

### Anti-biofilm Activity of Biocompatible Hydrogel Coatings

**Figure 6:**
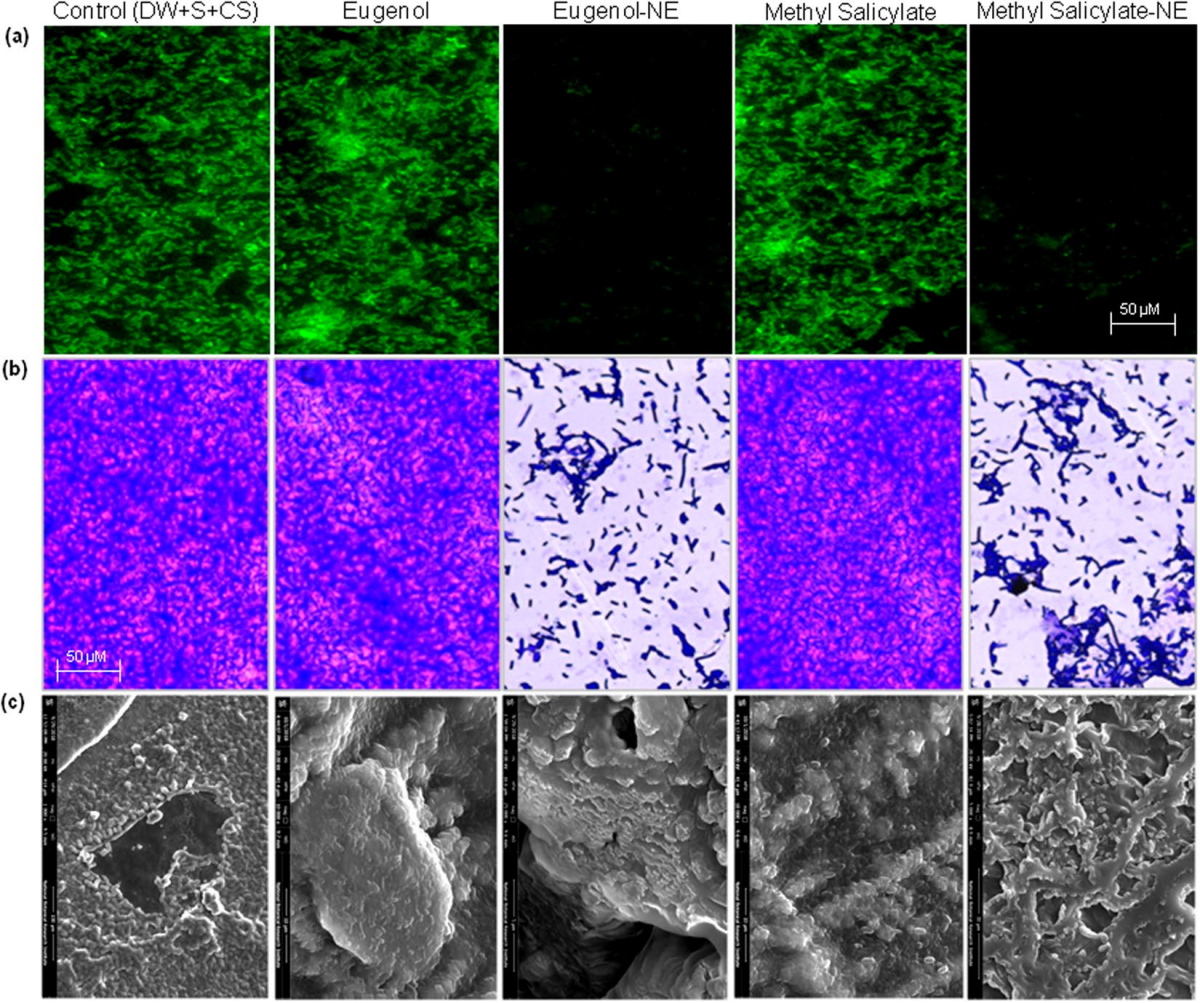
Inhibition of biofilm formation by ECOH cells exposed to hydrogel coatings containing eugenol-NE (0.001%) and methyl salicylate-NE (0.005%) for 7 days on solid surfaces, namely (**a**) glass, (**b**) plastic and (**c**) meat and measured by fluorescence microscopy, phase contrast microscopy and SEM, respectively. The mixture of DW, surfactant (S; Tween 80) and co-surfactant (CS; propylene glycol) in the ration of 94.7:0.2:0.1 was used as a solvent control.

**Figure 7:**
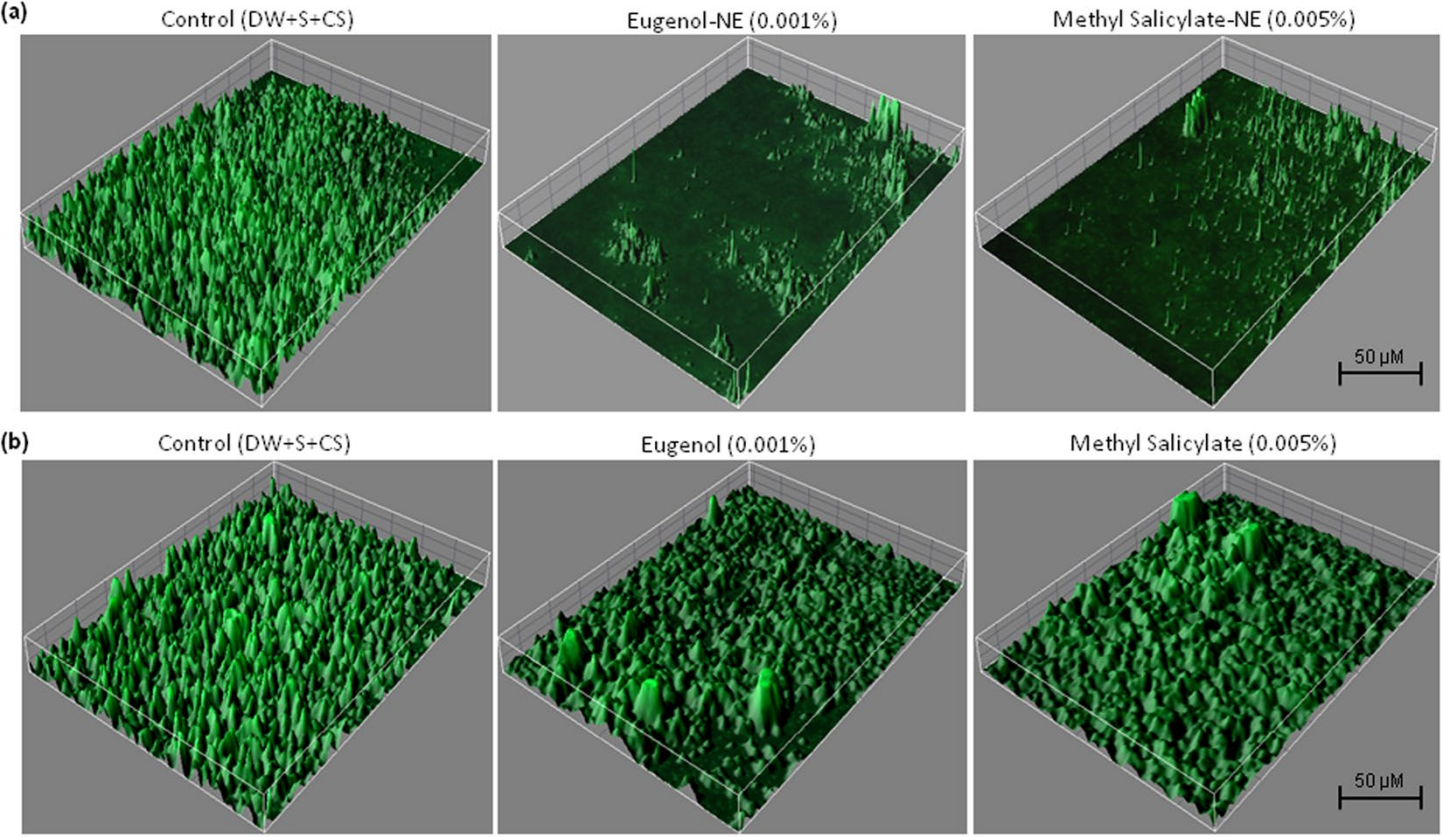
Anti-biofilm activity of hydrogel coatings containing (**a**) eugenol-NE and methyl salicylate-NE, (**b**) eugenol and methyl salicylate against ECOH on glass surfaces, measured by confocal laser microscopy. The mixture of DW, surfactant (S; Tween 80) and co-surfactant (CS; propylene glycol) in the ration of 94.7:0.2:0.1 was used as a solvent control.

**Figure 8:**
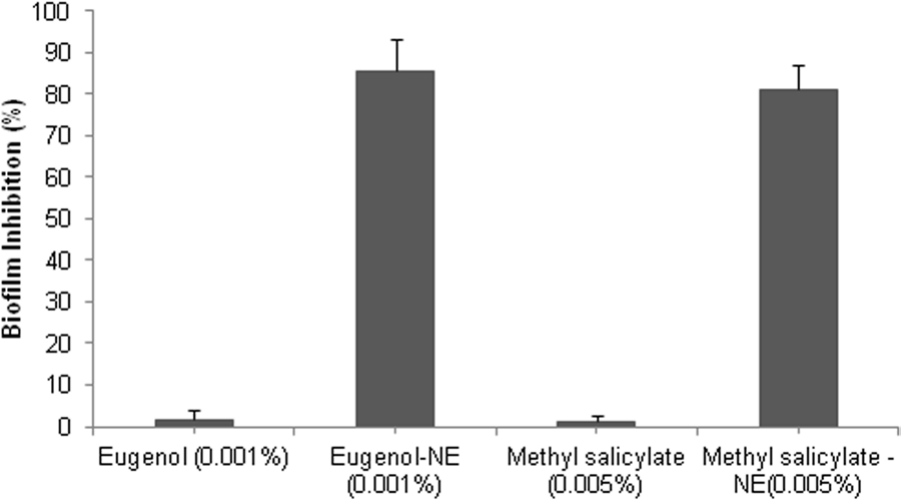
Anti-biofilm activity of hydrogel coatings containing eugenol-NE and methyl salicylate-NE on plastic surface against ECOH biofilm formation. The biofilm formation was measured at A_545_ using a ELISA plate reader through extraction of biofilm-bounded CV dye after treatment of 24 h. The results were expressed as arithmetic mean ± SE. The experiments were carried out at least thrice in independent experiments having three replicates.

To inhibit biofilm formation of ECOH on solid surfaces, prepared NEs of eugenol and methyl-salicylate were incorporated into a biocompatible hydrogel, prepared from 0.9% carbopol 940, a food grade polymer. As expected hydrogel coatings containing eugenol-NE (0.001%) and methyl-salicylate-NE (0.005%) noticeably reduced biofilm formation of ECOH on glass coverslips (Fig. 6a). COMSTAT analysis displayed that the NEs of eugenol and methyl-salicylate inhibit biofilm biomass, mean thickness, and substratum coverage by more than 80% (Fig. 7; Table S5). The hydrogel coatings inhibited biofilm formation on polystyrene plates by more than 80% as analysed by CV-staining (Fig. 6b) and ELISA (Fig. 8) assays.

As ECOH colonizes and replicates in the meat it has the ability to spoil these food items^14,36^ and the impact of eugenol-NE and methyl salicylate-NE loaded hydrogel coatings on ECOH biofilm formation on meat surface was studied. To prevent biofilm formation of ECOH on meat surfaces, the pieces were coated with hydrogel coatings containing eugenol-NE (0.001%) and methyl-salicylate-NE (0.005%) and subsequently inoculated with ECOH (2 × 10^10^ cfu/ml). After incubation for 24 h, the anti-biofilm activity of NEs was analysed by SEM. A remarkable anti-biofilm activity of eugenol-NE (0.001%) and methyl-salicylate-NE (0.005%) was observed (Fig. 6c). Interestingly, hydrogel coatings containing eugenol (0.001%) and methyl-salicylate (0.005%) failed to inhibit ECOH biofilm formation on glass, plastic and meat surfaces (Figs 6a–c, S9b, S10). The results indicate that biocompatible hydrogel coatings can be useful to prevent ECOH biofilm formation on solid surfaces.

## Discussion

**Figure 9:**
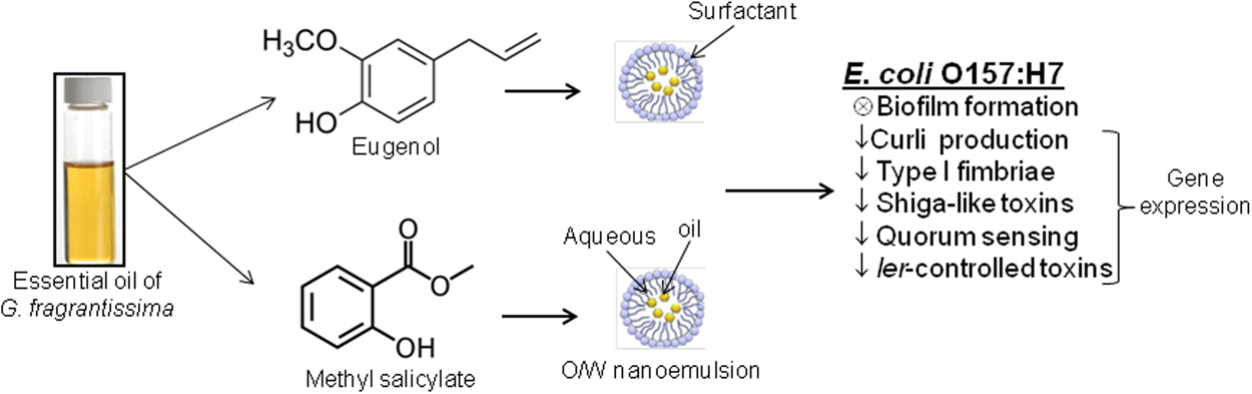
A schematic diagram explaining the mechanism of anti-biofilm and anti-virulence activities of NEs of *G*. *fragrantissima*.

*E*. *coli* infection is a major problem worldwide owing to the emergence of multi-drug resistance biofilms and due to the lack of effective therapy. The current investigation demonstrates that the stable NEs of *G*. *fragrantissima* EO and its bioactive compounds showed superior anti-biofilm property than pure EO against ECOH without altering its cell viability. The inhibition of biofilm formation could be an attractive option to inhibit cell growth by antibiotics because it does not enforce a selective pressure, and thus the risk of drug resistance is less likely to develop. Current investigation using the bioactive compounds of EO and their NEs proved the anti-biofilm activity of eugenol, identified in the EO of WGF and methyl salicylate, identified in both WGF as well as CGF OEs. Biochemical and transcriptional experiments presented evidence regarding the mechanism of biofilm inhibition and virulence reduction by eugenol-NE and methyl salicylate-NE (Fig. 9). Furthermore, NEs of eugenol and methyl salicylate remarkably inhibited ECOH biofilm formation on solid surfaces such as glass, plastic and meat pieces.

Anti-biofilm and anti-virulence properties of eugenol-NE and methyl salicylate-NE were due to the suppression of LPS production, motility, fimbriae production and biofilm-related genes in ECOH^37,38^. It is well-documented that LPS production and fimbriae formation are major virulence factors which play a key role in biofilm formation. The bacterial strains develop different types of fimbriae, including curli fimbriae, type I fimbriae, pilus, F9 fimbriae, and other fimbrial proteins^39^. Eugenol is a volatile chemical of several plants belonging to the Lamiaceae, Lauraceae, Myrtaceae, and Myristicaceae families^29^. A large number of scientific studies suggests that eugenol exerts strong anti-biofilm potential and antimicrobial activity which was also tested in several experimental model systems^16^. Earlier studies have reported that the bioactive compounds of EOs such as eugenol, cinnamaldehyde, coumarin, and thymol reduce biofilm formation in *E*. *coli* through attenuation of curli fimbriae production^13,40,41^. Recently, a study revealed that NEs of EOs of cumin, pepper, and fennel inhibit biofilm formation and LPS production in *E*. *coli* and other food borne bacteria^14^. Therefore LPS and fimbriae inhibiting potential of plant EOs are already well documented and the inhibition by the NEs of bioactive compounds of EOs can be considered as a potential target for attenuation of ECOH biofilm formation.

To date, a number of EOs have been reported to suppress quorum sensing and biofilm formation in various pathogenic bacteria^42^. Previous studies have shown that eugenol possesses anti-biofilm activity against *Pseudomonas aeruginosa*, *E*. *coli*, *Staphylococcus aureus*, and *Listeria monocytogenes*^43–45^. The current study demonstrates for the first time that *G*. *fragrantissima* EOs as well as NEs of eugenol and methyl salicylate have anti-biofilm activity against ECOH, and elucidates the mechanisms of action involved. Moreover, methyl salicylate and its NE has been reported for the first time for anti-biofilm activity. Methyl salicylate and eugenol are used in topical analgesic oils, creams, perfumes, flavoring agents, and local antiseptic and anesthetic products^46^. Methyl salicylate is abundantly found in *G*. *fragrantissima* EO and eugenol is found in clove oil. The study suggests new application of *G*. *fragrantissima* EO to prevent ECOH biofilm formation on solid surfaces.

Shiga-like toxin (STX) is generated by ECOH for attaching to host epithelial cells that causes the hemolytic-uremic syndrome. STX is encoded by LEE genes and is controlled by the *ler* gene. In the current investigation, the expression of *ler* and *ler*-regulated toxin genes (*espD*, *escJ*, *escR*, and *tir*) and curli genes (*csgABDFG*) was downregulated, when ECOH exposed to NEs of eugenol and methyl salicylate. Furthermore, NEs of eugenol and methyl salicylate also inhibited the expression of genes that regulate LPS production (*waaL*), quorum sensing (*luxS*, *luxR* and *tnaA*), and swarming (*fimA* and *fimH*) and swimming (*flhD*, *fliA*, and *motB*) motilities. The obtained data suggest that the NEs of eugenol and methyl salicylate can be used as potential inhibitors of biofilm formation and toxin production in LEE-encoding *E*. *coli* strains^47–49^.

In the current study, NEs of *G*. *fragrantissima* EOs and their bioactive chemicals namely, eugenol and methyl salicylate effectively inhibited the production of biofilm formation, pathogenicity factors, and expression of virulence genes in ECOH as compared to their bulk forms. In addition, hydrogel coatings containing eugenol-NE and methyl salicylate-NE effectively attenuated ECOH biofilm formation on plastic, glass, and meat surfaces. The obtained data suggests that the NEs of eugenol and methyl salicylate could be ideal candidates for the prevention of biofilm formation and virulence of ECOH on solid surfaces.

## Materials and Methods

### Plant Sample and Extraction

Fresh and healthy leaves of *G*. *fragrantissima* from Upper Shillong, Khasi Hills district, Meghalaya, North-eastern India (25°07″ 25°41″N Lat. & 91°21″ 92°09″E) were collected in August, 2018 under the scheme of Department of Biotechnology of India (Letter No. BT/01/17/NE/TAX/GAP-3439). The specimen was identified using local floras and the voucher specimen was deposited at the herbarium of the CSIR-National Botanical Research Institute, Lucknow, India. Collected leaves were washed thoroughly in tap water, followed by successive washings in DW. One kilogram of chopped leaves were subjected to hydro-distillation for 4 h using a Clevenger assembly following the procedure described in the Indian Ayurvedic Pharmacopeia Part-I, Volume-V. The amount of EO obtained from 1 kg of chopped fresh leaves of *G*. *fragrantissima* was to be 1.1% (w/v). To obtain pure EO, the collected EO was re-extracted with dichloromethane and water content was dried by adding anhydrous sodium sulphate power. The EO recovered from sodium sulfate through filtration and the pure EO was stored in a sealed amber-colored vial at a low temperature (4 ± 2 °C) until further analysis.

### Culture Conditions, Culture Media, Cell Growth Kinetics and Minimum Inhibitory Concentration (MIC) analysis

All experiments were carried out at 37 °C in nutrient agar (NA) and broth (NB) media, which were used to culture *E*. *coli* O157:H7 (ECOH; ATCC 43895). ECOH cells were initially streaked from glycerol stock on NA plates and incubated at 37 °C for 24 h. A fresh single colony was picked up and inoculated in NB medium for cultivation of ECOH. A stationary phase culture of ECOH having initial turbidity of 0.05 at A_600_ was inoculated in NB medium to perform biochemical and phenotypic assays. The EO of *G*. *fragrantissima* was procured from Amazon, USA. Other eugenol and methyl salicylate were obtained from Sigma-Aldrich (St. Louis, USA). For cell growth kinetics and minimum inhibitory concentration (MIC) analysis, turbidity was recorded at A_600_ using a UV-vis spectrophotometer (Thermo Fisher, USA). Growth kinetics assays were also performed against ECOH using NB medium to determine MIC and sub-MIC levels of EOs and NEs.

### NE Preparation and Characterization

Oil in water (O/W) NEs of *G*. *fragrantissima* leaves were prepared using EO (5%), S (Tween 80; 0.2%), CS (propylene glycol; 0.1%) and DW in the ratio of 5:0.2:0.1:94.7, respectively. The detailed methodology is provided in the Supplementary Methods section. The particle size of prepared NEs was measured using a particle size analyzer (MAL 1010294 Malvern, Worcestershire, UK). The fluctuation in the scattered light intensity, (Brownian motion) of the NE was measured by the dynamic light scattering (DLS) technique. To avoid multiple scattering effects, NEs were diluted with double DW. To determine the oil droplet dispersion in the NEs, size distribution curves were plotted as a function of intensity (%), Z-average average droplet size (d.nm), and PDI were calculated. Further, SEM was performed to determine the morphology and structure of the prepared NEs. To perform SEM, NEs were diluted 250-fold in with ultra-pure water and mounted on lamels and dried at room temperature. Samples were analyzed using a high-resolution Quanta-250 microscope (FEI, Netherland). TEM (JEM-1010 JEOL, USA) was carried out to determine the size and shape of droplets. For analysis, one drop of NE was negatively stained with phosphotungstic acid and placed on a copper grid. TEM images were obtained using a (JEM-1010 JEOL, USA) at an acceleration voltage of 80 kV. For the preparation of eugenol and methyl salicylate NEs, 3% of these compounds, 98.6% of water, 0.3% of Tween-80 and 0.1% of propylene glycol were used. The mixtures were sonicated using a 50 kHz Ultrasonic Processor (GEX 750, USA) under 750 W power output for 15 min in an ice-bath to prevent the evaporation of OEs. For the formation of ultrasonic waves, a 13 mm diameter ultrasound probe was used. To nullify heat energy generation during the ultrasonication process, the sample container was kept in an ice bath. The transparent and clear NEs were obtained and further subjected for characterization and stability analysis.

### Stability of NEs

The resistance of prepared NEs to centrifugation was assessed at room temperature by centrifuging NEs at 10,000 rpm for 20 min. Additionally, NEs (20 ml in glass tube) were stored at different temperatures (4 °C, −20 °C and 45 °C) and examined for phase separation or creaming.

### Biofilm Formation Assays

Static biofilm formation experiments were carried out in a 96-well polystyrene plate (Nunc, USA) using CV staining method, as previously described^50^. Briefly, stationary phase cells (0.3 ml) of enterohemorrhagic ECOH were inoculated in NB at initial turbidity of 0.05 OD at A_600_. The cells were grown with or without EOs and NEs at 37 °C without shaking. After incubation for 24 h, cells were washed thrice with DW to separate out non-adherent cells. The biofilms were stained with 0.5% aqueous solution of CV (Sigma-Aldrich) for 30 min at room temperature and washed thrice with DW. The bounded dye was extracted using 95% ethanol and recorded the OD at A_545_ using a microplate reader (BioRad, CA, USA). To normalize the measured values, the OD at A_545_ values were stabilized by the OD at A_595_.

Anti-biofilm activity of test samples was analyzed microscopically as described recently (Singh *et al*., 2015). Briefly, overnight ECOH cultures (1.0 OD_600_) were seeded on glass cover slides in two batches at 37 °C for 24 h. After washing three times with DW, slides were stained with CV (0.5%) and SYTO-9 (20 mM) for 15 min at room temperature. The CV-stained cells were analyzed by phase contrast microscope and SYTO-9 stained cells were analyzed at 480 nm (excitation) using a fluorescent microscope (Leica, Germany). To quantify biofilm formation, 20 fluorescence images were converted to grayscale through ImageJ software. To measure the biomass (μm^3^ per μm^2^), mean thicknesses (μm), and substratum coverage (%), COMSTAT biofilm software was used^51^.

### Anti-biofilm Formation Assessment by SEM

The overnight grew ECOH cells were treated with test samples for 24 h and then cells were washed thrice with DW, and fixed in 2.0% glutaraldehyde solution having pH 7.0 which is prepared in 0.1 M phosphate buffer. Cells were washed thrice in phosphate buffer saline and stained with saturated solutions of uranyl acetate and lead citrate. Inhibition of biofilm formation was examined by SEM (Quanta-250, FEI, Netherland) with an accelerating voltage of 10 kV.

### GC-MS Analysis

Chemical fingerprinting of wild and commercial EOs of *G*. *fragrantissima* were carried out by GC-MS using the model DSQ II (Thermo Scientific, USA) with a fused silica capillary column (30 m by 0.32 m i.d., the film thickness of 0.25 µm) and a TR 50 mass spectrometer. To perform GC-MS, an electron ionization system was maintained at 70 eV and the flow rate of the carrier gas (helium) was kept at 1 ml/min. The injector and detector lines were set at 250 °C and 290 °C, respectively. The initial oven temperature of 50 °C was kept for 1 min and subsequently raised to 310 °C gradually increased by 20 °C/min with a holding time at 310 °C for 15 min. A postrun temperature of 70 °C for 10 min was adequate for the subsequent insertion. The diluted samples (1:100 (v/v) were injected manually in split-less mode. The relative percentage of compounds were estimated by normalizing peak areas. Identification of compounds was achieved by matching the experimental mass spectra to the mass spectra available in the NIST library.

### Measurement of Virulence Factors

To measure the LPS inhibition, NB with and without prepared eugenol-NE and methyl salicylate-NE were inoculated with an overnight culture of ECOH having OD 1.0 at A_600_ and kept at 37 °C for 24 h. The cells were removed by centrifugation at 8000 g for 20 min at 4 °C. To precipitate the dislodged LPS, the obtained supernatant was mixed with chilled ethanol (1:3 ratio) and incubated overnight at 4 °C. The mixture was centrifuged at 8000 g for 20 min to collect the precipitated LPS and re-dissolved in 1 ml of DW. LPS content was quantified using the phenol-sulfuric acid method to determine the total carbohydrate and the absorbance was recorded at A_490_. Glucose was used as the working standard in the range of 0–100 µg dose.

The anti-virulence activity of eugenol-NE and methyl salicylate-NE on flagellar function was studied by quantifying ECOH swimming and swarming motilities. Swimming motility was assessed using NB media supplemented with 0.3% agar power and swarming motility was determined using NB media supplemented with 0.5% agar. The NEs were mixed to motility agar media. ECOH was cultured when OD reached 1.0 at A_600,_ motility plates were inoculated with 1 μl aliquots of cultures under aseptic conditions. After incubation for 24 h, the sizes of swimming halos were measured. Inhibition of curli fimbriae formation was assessed by NA plate assay using Congo red dye^52^ and SEM^53^. To determine curli fimbriae formation, NA plate assay was applied. Briefly, NA media contained NEs, 20 μg/ml of Congo red (Sigma) and 10 μg/ml of Coomassie brilliant blue (Sigma). The plates were inoculated with overnight grown ECOH culture, incubated at 37 °C for 24 h, and evaluated for curli fimbriae formation. The same protocol was followed, except NB medium was used in place of NA in 14-ml round bottom tubes to quantify the curli fimbriae formation. After 24 h of incubation, cultures of ECOH were centrifuged at 10,000 g for 30 min and recorded the OD of the supernatant at A_490_. Also, SEM was used to examine the fimbriae formation. Briefly, stationary phase cultures of ECOH were incubated in NB media at 37 °C for 2 h with agitation at 250 rpm, and subsequently re-incubated for 2 h at the same temperature with or without eugenol-NE and methyl salicylate-NE under shaking condition. The cultures were immediately fixed with 2.0% glutaraldehyde and formaldehyde and then cells were harvested using a 0.22 μm nylon filter (Genetix, India). The small squares of the filter (0.5 × 0.5 mm) were prepared and post-fixed in sodium phosphate buffer, osmium, ethanol, and isoamyl acetate solutions. The filters were critical-point dried and analyzed by SEM at 20 kV.

### qRT-PCR

The stationary culture of ECOH having a starting OD_600_ of 0.05 was inoculated in 30 ml of NB in 250 ml conical flasks containing NEs and incubated at 37 °C with shaking at 250 rpm for 24 h. The QIAzol Lysis Reagent (QIAGEN) was used to isolate total RNA as per the manufacturer’s protocol. Total RNA (1 μg) was used to synthesize cDNA using a PTC 200 thermocycler (MJ Research, Inc., Waltham, MA, USA). qRT-PCR was applied to examine the transcription levels of targeted genes. The reaction mixture contained 2 μL cDNA, 10 μL 2X SYBR master mix (Applied Biosystems, USA), 150 nM each primer, 0.4 μL reference dye, and RNA free water to make up 20 μL total volume. The analysis was carried out at 50 °C for 50 min followed by denaturation at 95 °C for 10 s, pursued by annealing at 55 °C for 10 s and extension at 60 °C for 35 s. To verify the absence of non-specific amplicons, a dissociation analysis was also performed. The relative fold expression change was determined using the CT method. Gene-specific primers were designed using Primer 3 (v 0.4.0) (Table S6). The *rrsG* gene was used as an internal control (housekeeping gene). ABI StepOne RT-PCR System (Applied Biosystems) was used for two independent cultures.

### Preparation of Hydrogel-based Surface Coatings with Anti-biofilm NEs

For the preparation of hydrogel-based coatings, a biodegradable carbopol 940 (HiMedia, India) was used as previously described^54^. Precisely weighed quantity of carbopol 940 (0.9% w/v) was disseminated in DW maintained at 40 °C temperature with constant mixing using mechanical stirrer at 1200 rpm for 30 min. Eugenol or methyl salicylate or their NEs were added to the gel-base and well stirred. The pH of resulting hydrogels was adjusted to 6.0 using triethanolamine and blended slowly until a clear gel was obtained. NE-loaded hydrogels (50 mg) were applied to glass and plastic surfaces to prepare a 0.7~0.8 cm diameter coatings. The plates were subsequently air-dried for 12 h and surface sterilized by UV radiation treatment for 5 h. To provoke biofilm formation on NE-contained hydrogel-coated surfaces, ECOH cells (4 × 10^7^ CFU/ml) were inoculated along with NB medium and incubated at 37 °C for 7 days. Planktonic cells were eliminated through washing with PBS thrice, biofilm cells were stained with SYTO-9 and analyzed by a fluorescence microscopy using the procedure as described in the biofilm formation assay section. The meat (chicken) pieces were also coated with NE-loaded hydrogels for 2 h, followed by UV sterilization for 4 h. The pieces were inoculated with ECOH cells (4 × 10^7^ CFU/ml) for 7 days under aseptic conditions and then small sections were prepared and analyzed by SEM to examine the anti-biofilm activity of the prepared NEs.

### Statistical analysis

All data were expressed as arithmetic mean ± standard error (SE). All the experiments were carried out at least thrice in independent experiments each having three replicates. Student’s t-test was used to compare the groups. Statistical significance was set at P < 0.05.

## Supplementary information

Supplementary Information
