# Supplementary material for: Nanoemulsion-loaded hydrogel coatings for inhibition of bacterial virulence and biofilm formation on solid surfaces
Source: Sci Rep. 2019 Apr 24;9:6520. doi: 10.1038/s41598-019-43016-w (PMC6482171; doi:10.1038/s41598-019-43016-w)
Supplement: Supplementary file 1 — Supplementary Information [file 41598_2019_43016_MOESM1_ESM.pdf]

Supplementary Information

Nanoemulsion-loaded hydrogel coatings for inhibition of bacterial virulence and biofilm formation on solid surfaces

Prateeksha G, Saroj Kanta Barik and Brahma Nand Singh

Herbal Nanobiotechnology Lab, Pharmacology Division, CSIR-National Botanical Research Institute, Lucknow-226001, India

Supplementary Tables

Table S1. Stability studies of prepared NEs

NE	Stability up to 30 days				Stability up to 60 days				Stability up to 90 days			
	Centrifugation[a]	4 ^{0}C	-20 ^{0}C	45 ^{0}C	Centrifugation[a]	4 ^{0}C	-20 ^{0}C	45 ^{0}C	Centrifugation[a]	4 ^{0}C	-20 ^{0}C	45 ^{0}C
CGF-NE	√	√	√	√	√	√	√	√	√	√	√	√
WGF-NE	√	√	√	√	√	√	√	√	*	*	*	*

√ Neither phase separation nor creaming; * Partial phase separation and creaming
[a]Centrifu-gation for 20 min at 10,000 rpm at 25 ^{0}C

Table S2. MIC of CGF-EO, WGF-EO, CGF-NE and WGF-NE

Treatment	[a]MIC (%)
CGF-EO	0.5±0.028
WGF-EO	0.3±0.051
CGF-NE	0.1±0.017
WGF-NE	0.025±0.0032

[a]Minimum inhibitory concentration (MIC) was calculated using growth curve analysis. MIC is determined as the lowest concentration that exhibited complete inhibition of visible growth of the bacteria. DMSO was used as a solvent control for CGF-EO and WGF-EO, whereas the mixture of DW, surfactant (S; Tween 80) and co-surfactant (CS; propylene glycol) in the ration of 94.7:0.2:0.1 were used as a solvent control for CGF-NE and WGF-NE.

Data represent mean ± SE of six different experiments.

Table S3. MIC and sub-MIC concentrations of eugenol and methyl salicylate[a]

Treatment	Concentration (%)
**MIC**	
Eugenol	0.05±0.002
Methyl salicylate	0.25±0.03
**Sub-MIC**	
Eugenol	0.005±0.0004
Methyl salicylate	0.025±0.0017

[a]Minimum inhibitory concentration (MIC) and sub-MIC was calculated using growth curve analysis.
DMSO was used as a solvent control.
Data represent mean ± SE of six different experiments.

Table S4. MIC of eugenol-NE and methyl salicylate-NE[a]

Treatment	Concentration (%)
**MIC**	
Eugenol-NE	0.005±0.0007
Methyl salicylate-NE	0.025±0.0043

[a]Minimum inhibitory concentration (MIC) was calculated using growth curve analysis.
Data represent mean ± SE of six different experiments. The mixture of DW, surfactant (S; Tween 80) and co-surfactant (CS; propylene glycol) in the ration of 94.7:0.2:0.1 were used as a solvent control.

Table S5. Antibiofilm activity of eugenol-NE and methyl salicylate-NE using COMSTAT analysis

Treatment	Volume/Area ($\mu m^3\ \mu m^{-2}$)	Mean thickness (μm)	Substratum coverage (%)
Control	19.2±1.83	16.9±1.44	94±8.52
Eugenol (0.0005%)	18.9±1.99	16.6±1.82	93±7.74
Eugenol-NE (0.00051%)	2.77±0.41	2.13±0.46	9.66±0.83
Methyl salicylate (0.0025%%)	19.1±1.83	16.9±1.59	94.4±9.92
Methyl salicylate -NE (0.0025%)	3.45±0.41	2.42±0.22	12±1.15

The mixture of DW, surfactant (S; Tween 80) and co-surfactant (CS; propylene glycol) in the ration of 94.7:0.2:0.1 were used as a solvent control.
Data represent mean ± SE of six different experiments.

Table S6. List of primers of *E. coli*

Gene	Forward (5'-3')	Reverse (5'-3')	Product length
escJ	ACGAAAGTCAACCCTCCTCAG	ATCTGAATGACCGATGGTGCT	79
escR	ACCTAGCCAGTTAGTGGCATC	AACCTCTGGCGAGCTGATGA	187
espD	ATTAGTGACGCCCTCTGCTG	ATTTGCTGAGGGTCAACGGT	150
rrsG	ACTGAGACACGGTCCAGACT	TGCGCTTTACGCCCAGTAAT	259
csgF	CTCAGGCCATCCAGTCACAA	AAACCCGAAACCTGGATGGT	178
fim A	CACGACGGTTAATGGTGGGA	GTCCAGGATCTGCACACCAA	310
flhD	ACCTCCGAGTTGCTGAAACA	TGCCAGCTTAACCATTTGCG	168
fliA	CGGAAACTGAGGTAGCGGAA	CTCCAGCACTGCACCAATCT	299
qseB	GATTGGCGACGGCATCAAAA	TCAGGATCAGTACCGGCTCA	203
fimC	GGTGGAAAATGCCGATGGTG	AAACTTTCCCGGTCCTGTGG	138
tnaA	TCACCCGCGAAACCTACAAA	AGCCAGTCCAGATTCATGCC	235
bcsA	TTTTCGACTGCGACCACGTA	TAGAACAGCGTGCCTTCGTT	175
csgD	CGCGGCGAATGCTACTTTAC	TCGTTATTAGACGCGCCGAT	149
csgB	AACCTTCTTTGCAGGCGACA	ATGTCGCGGTACGGGTAATC	241
csgG	GCCTGATGTCGGCTATCGAA	TGTCATTCTGCCGTTCTGCT	102
csgA	ATCTGACCCAACGTGGCTTT	CTGAGTTACGTTGACGGTGGA	146
fimD	GGGGAGGCGATGGTAATAGC	AACAAGCACCACCGTATCGT	194
fimH	GCAGTTCTCCTACAGCGGTT	AATGACGACCGAACCAACGA	167
stx1	TTACAGCGTGTTGCAGGGAT	CAGACTGCGTCAGTGAGGTT	112
stx2	GCGACGCCTGATTGTGTAAC	CCCGTAATTTGCGCACTGAG	143
luxS	TTCTTCGTTGCTGTTGATGC	TGGAAGACGTGCTGAAAGTG	152
luxR	CTCGAACGCCTGAACTTTTC	TGGAAGACGTGCTGAAAGTG	231
ecpA	TGCTGACGTAACAGCTCAGG	CCTCGATAGCCACGTCAAAT	164
ecpR	ACATCTGGTCTCCCCATGAC	TACCGCGGATAACCATTCAT	179
tir	CGTCTTCCGTTCACGGTACT	ACTTCTGATCCTGGCCACTG	154
Z2200	TTG CGA TAC CAA TGT TGC AT	TGG AAC GGA ATG GTA TTG GT	141
ler	CGA CCA GGT CTG CCC TTC T	GCG CGG AAC TCA TCG AAA	174

Supplementary Figures

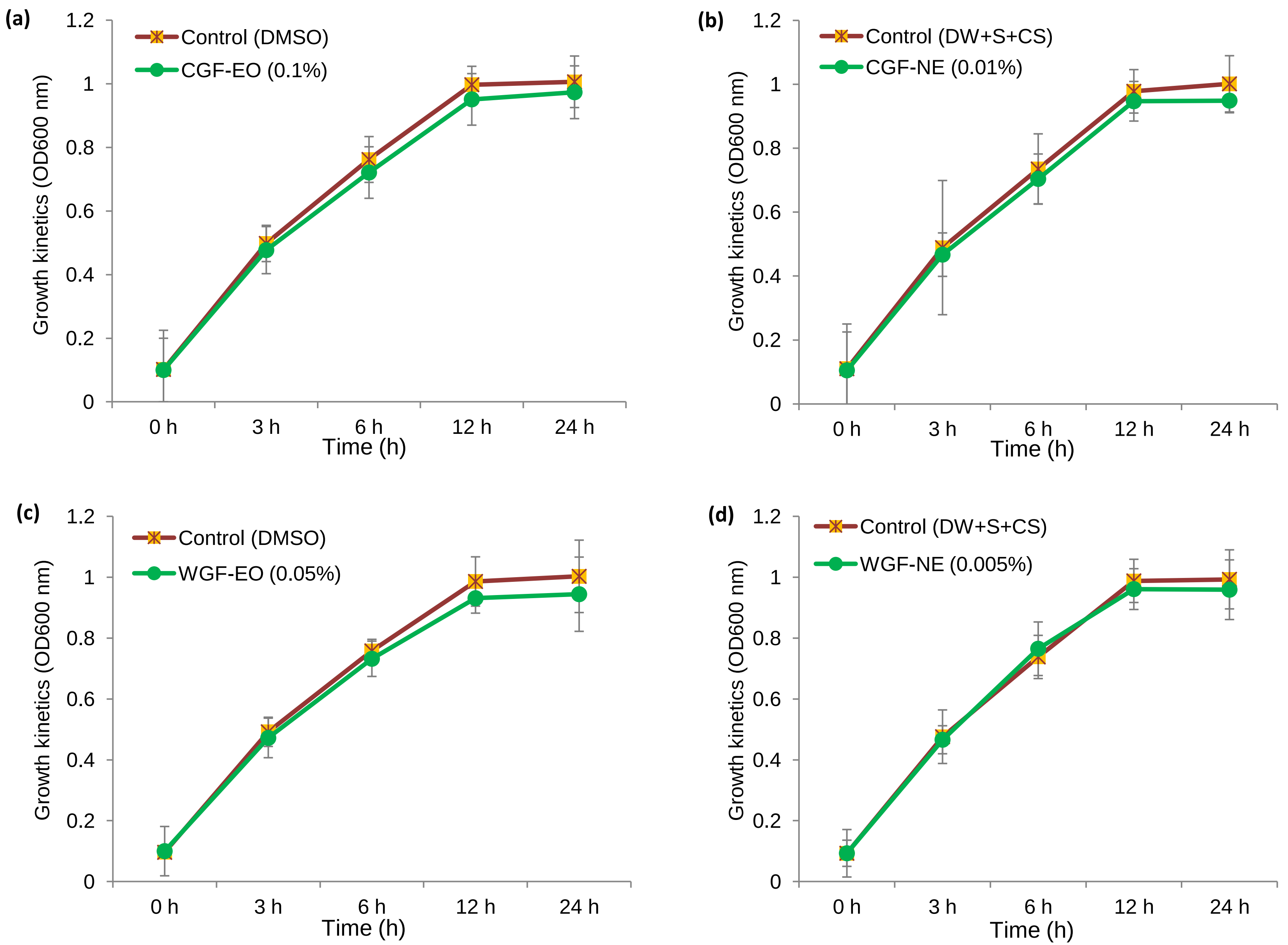

Figure S1. Growth kinetics of ECOH in the presence or absence of CGF-EO (a), CGF-NE (b), WGF-EO (c) and WGF-NE (d) with indicated concentrations for the period of 24 h. DMSO was used as a solvent control for CGF-EO and WGF-EO, whereas the mixture of DW, surfactant (S; Tween 80) and co-surfactant (CS; propylene glycol) in the ration of 94.7:0.2:0.1 was used as a solvent control for CGF-NE and WGF-NE.

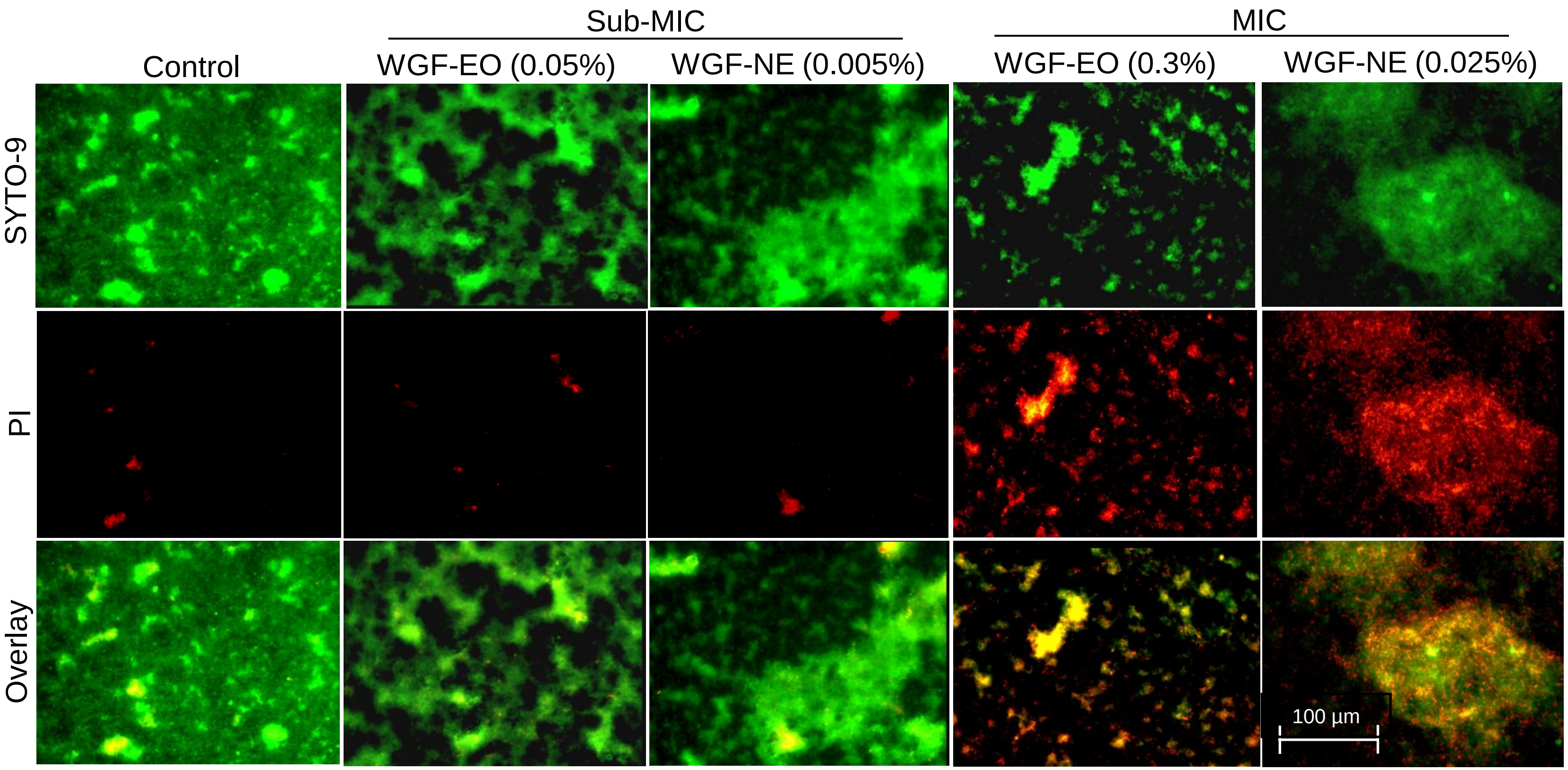

Figure S2. Cell viability measurement of ECOH. Cells were exposed to mentioned test samples with indicated concentrations for 24 h. Cells were analyzed by fluorescence microscope using bacterial cell viability kit. The mixture of DW, surfactant (S; Tween 80) and co-surfactant (CS; propylene glycol) in the ration of 94.7:0.2:0.1 was used as a solvent control.

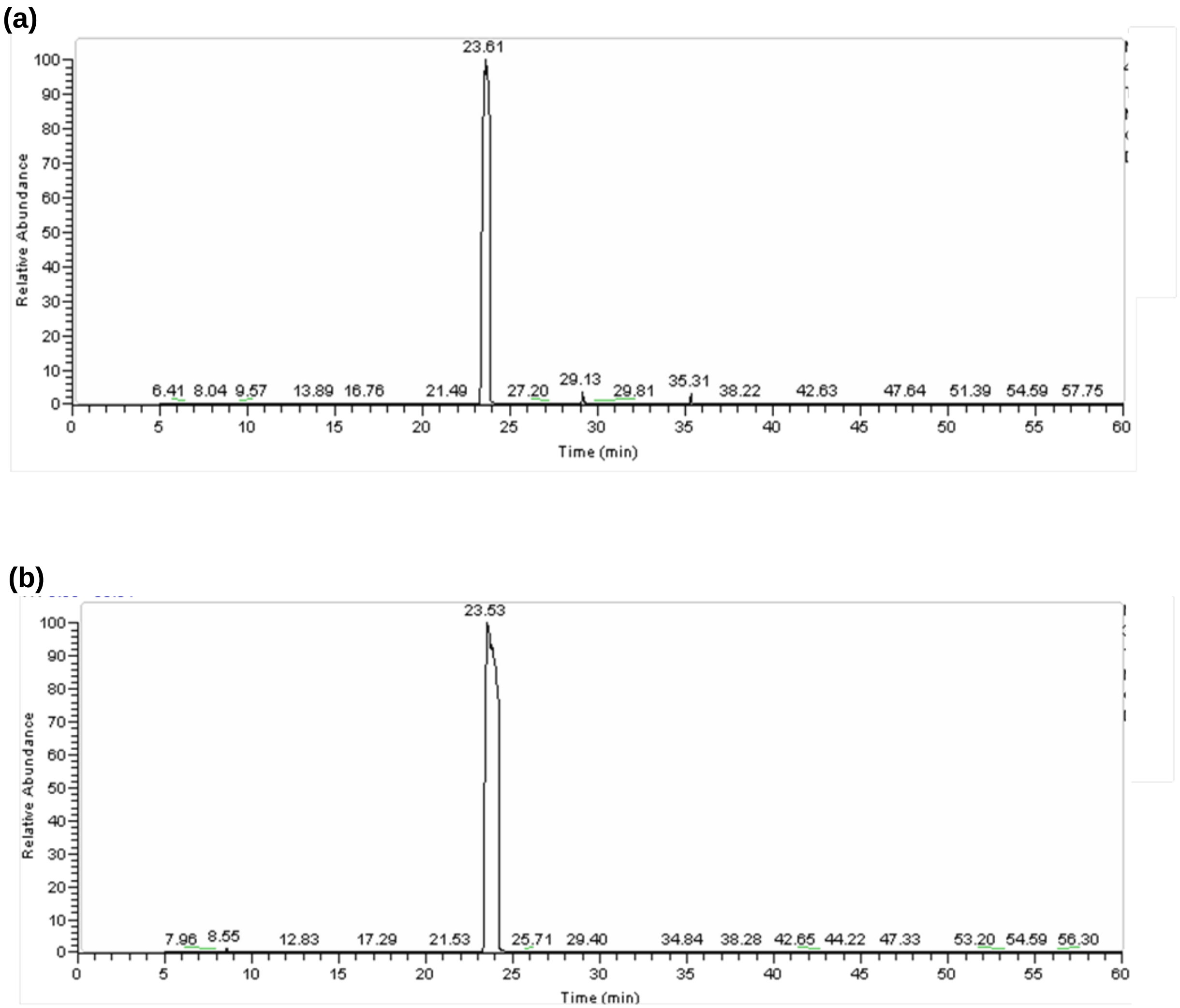

Figure S3. GC-MS chromatograms of WGF-EO (a) and CGF-EO (b) .

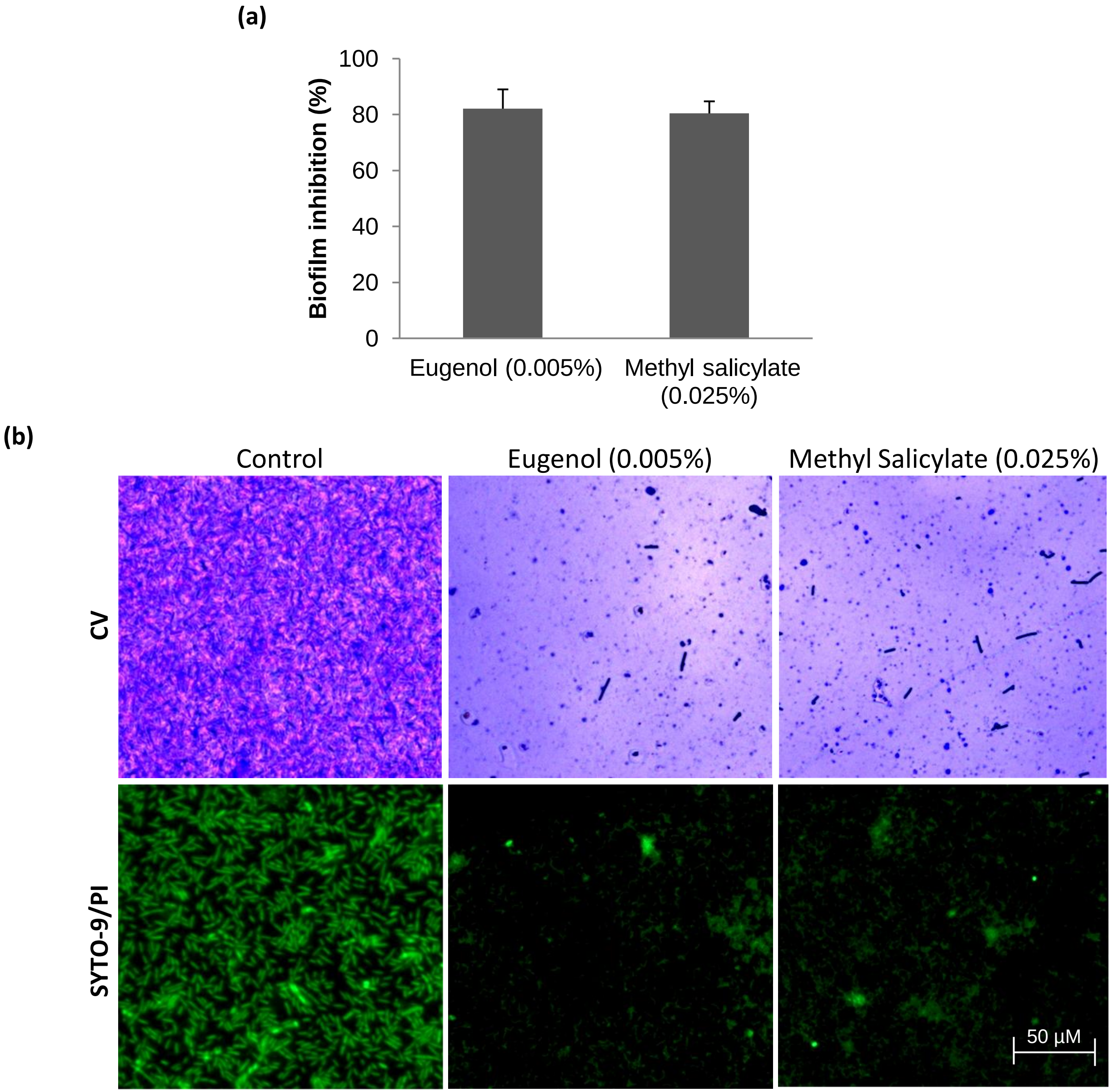

Figure S4. Impact of eugenol and methyl salicylate on ECOH biofilm formation. (a) The formation of biofilm by ECOH was measured using CV staining assay in the presence of major bioactive compounds after culture for 24 h in 96-well plates. Biofilm formation by ECOH in the presence of essential oils (0.005%) was also confirmed through (a) CV cell staining by phase contrast microscopy and (b) bacterial cell viability kit by fluorescence microscopy. DMSO was used as a solvent control.

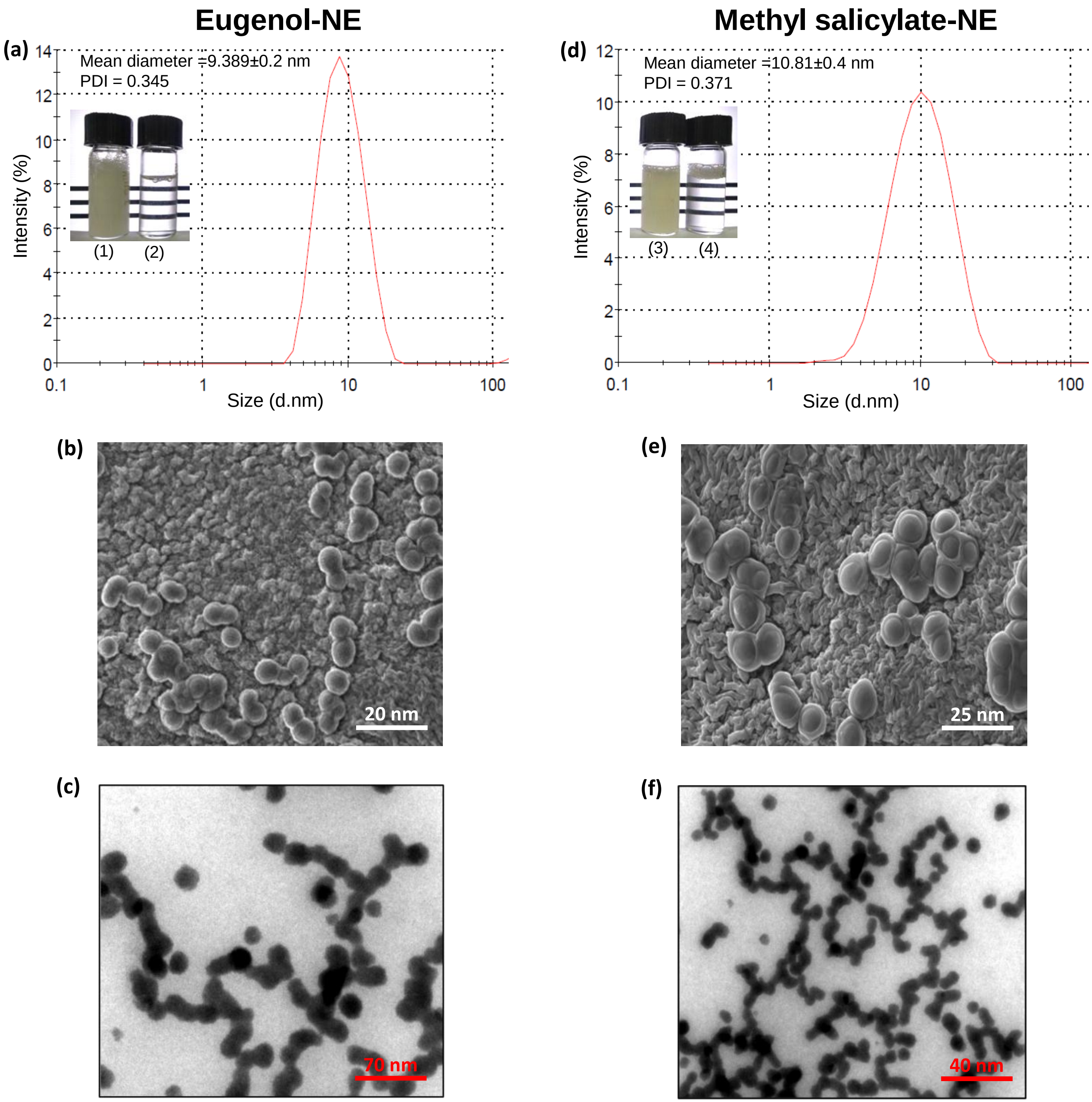

Eugenol-NE
Methyl salicylate-NE
(a)
Mean diameter =9.389±0.2 nm
PDI = 0.345
Intensity (%)
Size (d.nm)
(1)
(2)
(d)
Mean diameter =10.81±0.4 nm
PDI = 0.371
(3)
(4)
(b)
20 nm
(e)
25 nm
(c)
70 nm
(f)
40 nm

Figure S5. Characterization of NEs. DLS particle size distribution of (a) eugenol-NE and (d) methyl salicylate-NE. The insets depict the emulsion of eugenol (1), eugenol-NE (2), emulsion of methyl salicylate (3) and methyl salicylate-NE (4). SEM images of (b) eugenol-NE and (e) methyl salicylate-NE. TEM images of (c) eugenol-NE and (f) methyl salicylate-NE.

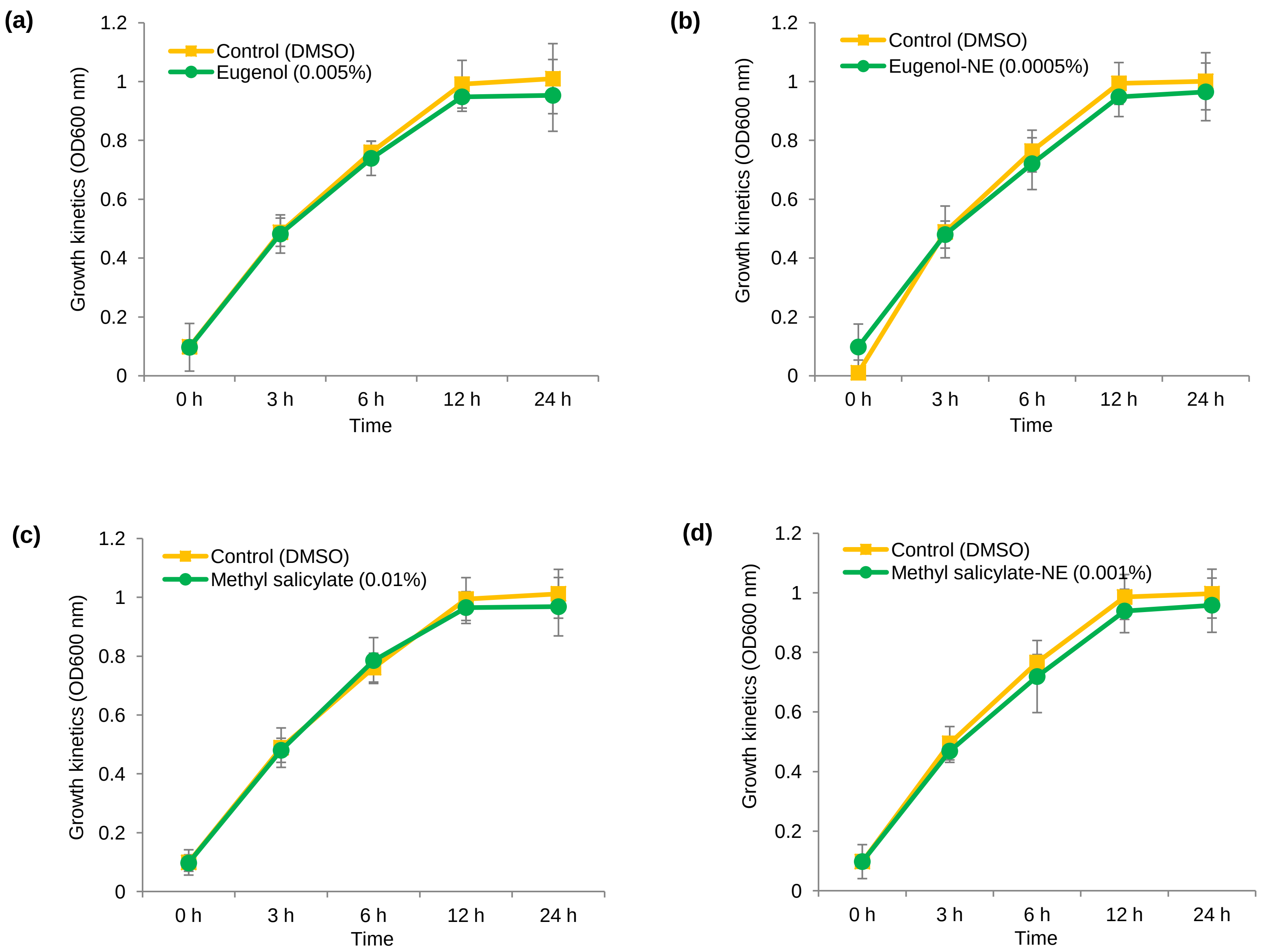

Figure S6. Growth kinetics of ECOH in the presence or absence of eugenol (a) eugenol-NE (b), methyl salicylate (c) and methyl salicylate-NE with indicated concentrations for the period of 24 h. DMSO was used as a solvent control for CGF-EO and WGF-EO, whereas the mixture of DW, surfactant (S; Tween 80) and co-surfactant (CS; propylene glycol) in the ration of 94.7:0.2:0.1 was used as a solvent control for CGF-NE and WGF-NE.

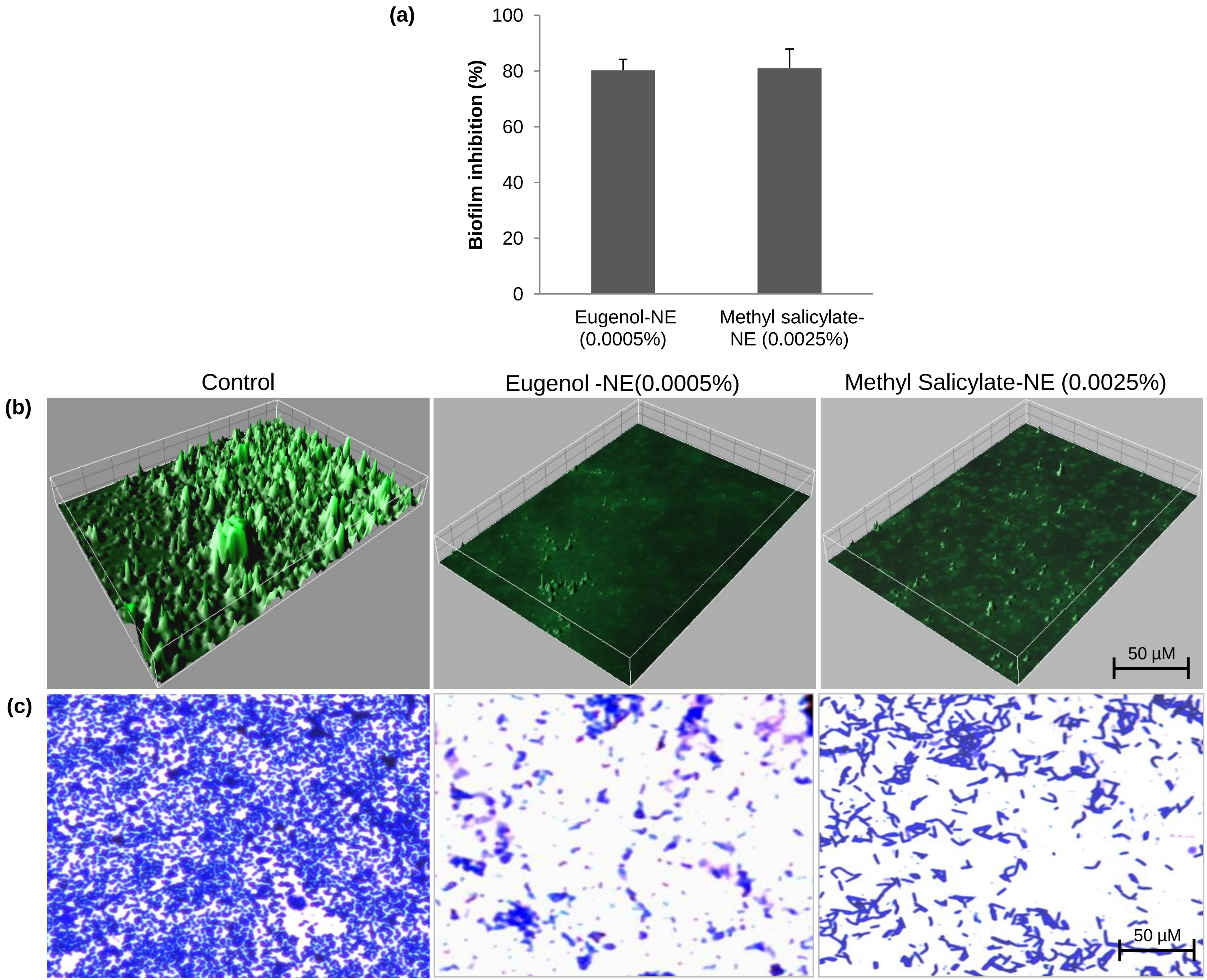
(a)
100
80
60
40
20
0
Biofilm inhibition (%)
Eugenol-NE (0.0005%)
Methyl salicylate-NE (0.0025%)
Control
Eugenol -NE(0.0005%)
Methyl Salicylate-NE (0.0025%)
(b)
50 µM
(c)
50 µM

Figure S7. Anti-biofim activity of eugenol-NE and methyl salicylate-NE. Inhibition of biofilm in the presence of NEs was measured after treatment of 24 h by ELISA in 96-well plates (a), confocal laser microscopy (b) and CV cell staining (c) techniques. The mixture of DW, surfactant (S; Tween 80) and co-surfactant (CS; propylene glycol) in the ration of 94.7:0.2:0.1 was used as a solvent control.

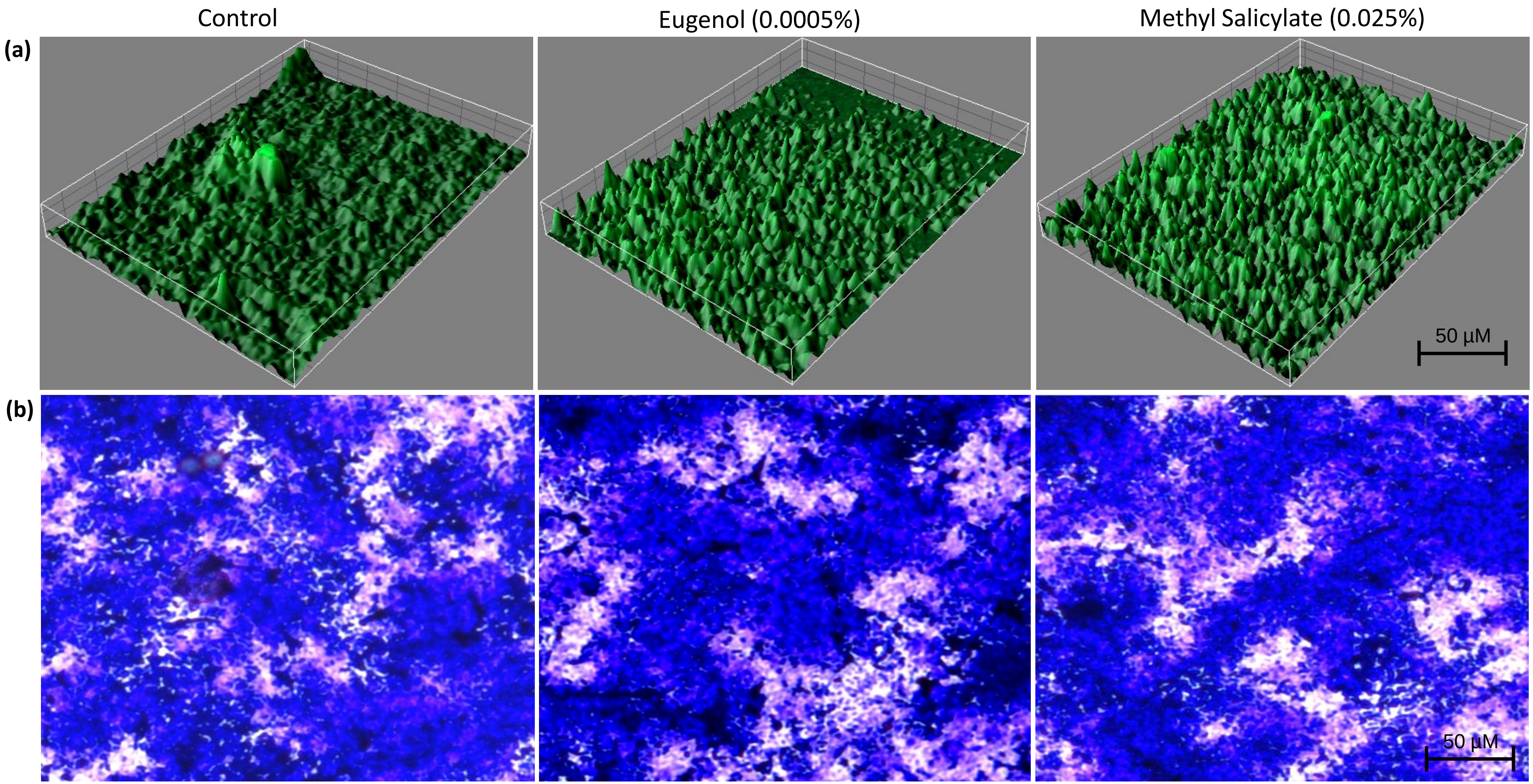

Figure S8. Biofim inhibition of ECOH by eugenol and methyl salicylate, measured by (a) confocal laser microscopy and (b) CV cell staining technique. DMSO was used as a solvent control.

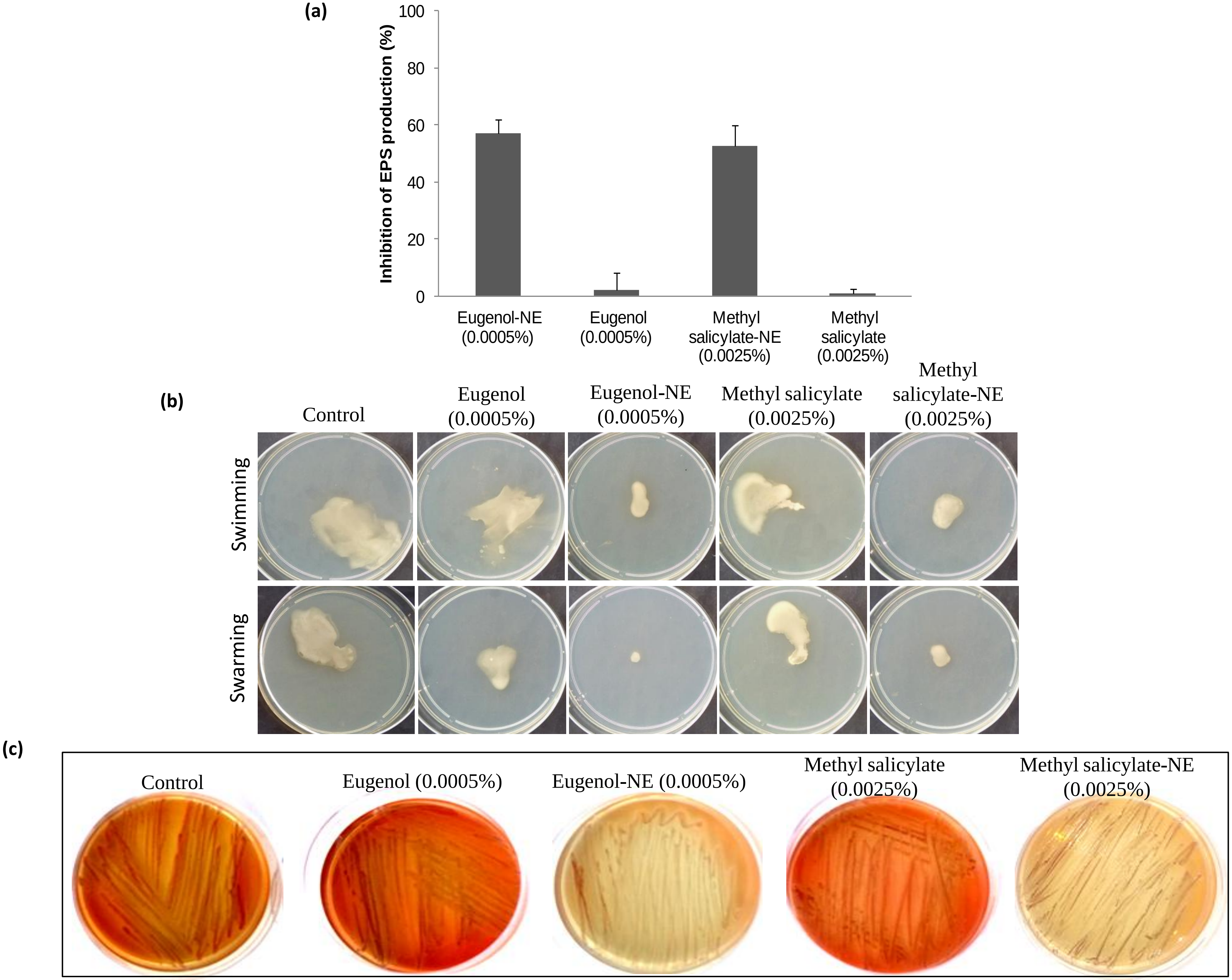
(a)
100
80
60
40
20
0
Inhibition of EPS production (%)
Eugenol-NE (0.0005%)
Eugenol (0.0005%)
Methyl salicylate-NE (0.0025%)
Methyl salicylate (0.0025%)
(b)
Control
Eugenol (0.0005%)
Eugenol-NE (0.0005%)
Methyl salicylate (0.0025%)
Methyl salicylate-NE (0.0025%)
Swimming
Swarming
(c)
Control
Eugenol (0.0005%)
Eugenol-NE (0.0005%)
Methyl salicylate (0.0025%)
Methyl salicylate-NE (0.0025%)

Figure S9. Effect of eugenol-NE and methyl salicylate-NE on the production of virulence factors, namely (a) EPS production, (b) swimming-swarming motilities, and (c) fimbriae production in ECOH cells treated for 24 h. The mixture of DW, surfactant (S; Tween 80) and co-surfactant (CS; propylene glycol) in the ration of 94.7:0.2:0.1 was used as a solvent control.
